# “Stockpile” of Slight Transcriptomic Changes Determines the Indirect Genotoxicity of Low-Dose BPA in Thyroid Cells

**DOI:** 10.1371/journal.pone.0151618

**Published:** 2016-03-16

**Authors:** Immacolata Porreca, Luisa Ulloa Severino, Fulvio D’Angelo, Danila Cuomo, Michele Ceccarelli, Lucia Altucci, Elena Amendola, Angela Nebbioso, Massimo Mallardo, Mario De Felice, Concetta Ambrosino

**Affiliations:** 1 IRGS, Biogem, Via Camporeale, 83031, Ariano Irpino, Avellino, Italy; 2 University of Trieste, PhD School of Nanotechnology, Piazzale Europa 1, 34127, Trieste, Italy; 3 Department of Science and Technology, University of Sannio, Via Port’Arsa 11, 82100, Benevento, Italy; 4 Department of Biochemistry, Biophysics and General Pathology, Second University of Naples, Via L. De Crecchio 7, 80138, Napoli, Italy; 5 Molecular Medicine and Medical Biotechnologies, University of Naples “Federico II”, via Pansini 6, 80131, Napoli, Italy; 6 IEOS-CNR, Via Pansini 6, 80131 Napoli, Italy; St. Georges University of London, UNITED KINGDOM

## Abstract

Epidemiological and experimental data highlighted the thyroid-disrupting activity of bisphenol A (BPA). Although pivotal to identify the mechanisms of toxicity, direct low-dose BPA effects on thyrocytes have not been assessed. Here, we report the results of microarray experiments revealing that the transcriptome reacts dynamically to low-dose BPA exposure, adapting the changes in gene expression to the exposure duration. The response involves many genes, enriching specific pathways and biological functions mainly cell death/proliferation or DNA repair. Their expression is only slightly altered but, since they enrich specific pathways, this results in major effects as shown here for transcripts involved in the DNA repair pathway. Indeed, even though no phenotypic changes are induced by the treatment, we show that the exposure to BPA impairs the cell response to further stressors. We experimentally verify that prolonged exposure to low doses of BPA results in a delayed response to UV-C-induced DNA damage, due to impairment of p21-Tp53 axis, with the BPA-treated cells more prone to cell death and DNA damage accumulation. The present findings shed light on a possible mechanism by which BPA, not able to directly cause genetic damage at environmental dose, may exert an indirect genotoxic activity.

## Introduction

In recent years, concerns about the effects of bisphenol A (BPA) on human health have been increasing in view of its widespread presence in human tissues and body fluids [1], with serum concentrations varying from 0.2 to 1.6 ng/ml (nanomolar range) [2]. Ingestion and transdermal absorption/inhalation are thought to be the primary and secondary routes of exposure in humans, respectively [2, 3]. Exposure is mainly due to BPA monomers leaching from BPA polymers present in food and water contact materials.

Epidemiological and experimental studies suggest a relationship between BPA exposure and different disease outcomes or developmental disorders [4, 5] as a result of BPA interference with hormonal signalling, above all with estrogen receptors [6].

Among endocrine organs, the thyroid gland is highly susceptible to environmental pollutants. BPA interference with thyroid hormone (TH) signalling [7, 8] may pose a hazard to human health as these hormones regulate a variety of biological processes associated with metabolism, energy provision, development, somatic growth and reproduction. Several lines of evidence suggest that BPA exposure might be associated with thyroid dysfunction, although complex and contradictory outcomes have been reported [9–13]. Recently, an inverse relation has been reported between BPA urinary level of the pregnant women and the thyrotropin (TSH) cord serum among girls in the prospective cohort of the HOME Study (2003–2006, Cincinnati, Ohio), although none differences in thyroid hormones has been detected in cord serum of newborns in the same study [14]. A direct effect of BPA on thyroid gene transcription has been studied. In experiments conducted on immortalized rat thyrocytes and in zebrafish embryos it has been shown that exposure to environmental doses of BPA increases the cellular level of transcripts involved in TH biosynthesis, in particular the *sodium iodide symporter* (NIS), *TSH-receptor* (TSH-R) and *thyroglobulin* (Tg). Furthermore, it increases the cellular content of their upstream regulators *Pax8* and *Nkx2-1* [15].

The incidence of thyroid cancer is increasing and it is thought to be linked to environmental carcinogenic factors [16]. Increased TSH levels and oxidative stress have been described as endogenous factors contributing to the rise in thyroid cancer incidence [16], and were also reported following exposure to BPA *in vivo* [9]. However, only sporadic data are available on the role of BPA in cancer development of other endodermal organs, i.e. prostate [17, 18]. Therefore, its involvement in thyroid carcinogenesis cannot be ruled out.

To characterize the effects of BPA exposure on thyrocytes as well as its mechanisms of toxicity we applied a toxicogenomic approach. Transcriptome analysis technologies have been suggested for the identification of mechanisms of compound toxicity. Providing the view of the expression profiles of many hundreds of genes in a specific biological condition, they can help in the understanding the related phenotype and molecular changes. In addition, pathway analysis technologies allows for clustering of gene-expression data into relevant pathway maps based on their functional annotation and known molecular interactions.

Due to the complexity of thyroid physiology *in vivo*, we investigated the direct effects of an environmentally relevant dose of BPA (10^−9^ M) on an immortalized rat follicular thyroid cell line (FRTL-5), whose response to such low BPA dose had already been verified [15]. Our approach shed light on the unexplored and time-dependent mechanisms of low-dose BPA toxicity in thyrocytes. We observed dynamic changes in the transcriptional response along the time, sign of the adaptation of the cell response to the length of exposure. Our data indicate that BPA impaired cellular response to further injuries, such as UV-C-induced DNA damage.

The findings reported here suggest, for the first time, that BPA exposure may contribute to thyroid cell damage by impairing DNA repair efficiency, and, likely, to thyroid carcinogenesis in cooperation with other endogenous or external factors.

## Materials and Methods

### Chemical Reagents

BPA, purchased from Sigma-Aldrich, was dissolved in dimethyl sulfoxide at 500 mM and used at 10^−9^ M.

### Cell Culture, Treatment, UV-C Irradiation and Cell Cycle Analysis

FRTL-5 cells were provided by Prof. Di Lauro in 2011. They have been tested for cell growth dependence by TSH (population doublings and MTT assay) and for the TSH-dependent expression of the thyroid specific genes (western blot and qRT-PCR) [15]. The FRTL-5 cells were routinely tested for the above parameters. The cells were maintained in Coon’s modified F12 medium (EuroClone) supplemented with 5% newborn bovine serum (HyClone Laboratories) and six hormones, including 1 mU/ml TSH (Sigma-Aldrich), and 10 μg/ml insulin (Sigma-Aldrich), as previously described [19], and discarded every 12 passages (6 weeks). All the treatments were performed using BPA at a concentration of 10^−9^ M, a dose within the range of BPA levels in human blood [2].

The proliferation rate was determined by seeding the FRTL-5 cells into 100 mm plates (2x10^5^ cells), and counting them every 7 days. The growth curve was generated by calculating the population doubling level with the formula PDL = 3.32 (logX-logY)+S, where:

Y = cell number at starting time, X = cell number at the end of incubation time, and S = starting PDL (www.atcc.org).

For UV-C irradiation experiments, cells were continuously treated with BPA for 28 days to ensure a long term exposure and to obtain a stabilisation of the observed transcriptional effects. After BPA treatment, cells untreated (controls) or treated with BPA were differently seeded in 100 mm plates according to the treatment time point (6 hrs = 4x10^6^ cells; 24, 48, 72 hrs = 1.2x10^6^ cells; 96, 120 hrs = 5x10^5^ cells). After medium removal and PBS wash, untreated and BPA-treated FRTL-5 cells were UV-C irradiated or left unexposed. UV-C irradiation was performed at a dose of 2 J/m^2^ UV-C (254 nm wavelength, Stratalinker, Stratagene), as already reported [20]. After irradiation, the medium was added and cells were collected at specific time points (24, 48, 72, 96, and 120 hrs). A growth curve was generated as described above.

Cell cycle and cell death analyses were performed as previously reported [21].

### Measurement of Cellular ROS Production

FRTL-5 cells were treated with 10^−9^ M BPA for 1, 3, and 7 days. For ROS measurement cells were incubated with 10 μM 2′,7′-dichlorodihydrofluorescein diacetate (H2DCFDA) for 1 hr at 37°C, in the dark. H2DCFDA is a cell-permeable non-fluorescent probe that is de-esterified intracellularly and turns to highly fluorescent 2′,7′-dichlorofluorescein upon oxidation, allowing sensitive and rapid measurement of oxygen-reactive species. After incubation, the cells were collected in PBS and ROS levels measured by spectrofluorometer (485 nm excitation; 535 nm emission) at each specific time point.

### Microarray and IPA Analysis

Three independent samples were prepared for each condition for microarray analysis and hybridized. Microarrays were performed with the Affymetrix platform using GeneChip^®^Rat Gene 2.0 ST Arrays (Affymetrix). Gene expression profiling experiments and data analyses were conducted as described [22]. Briefly, all samples passed the quality control check and the reproducibility of replicates was assessed by PCA. Raw data were normalized using the RMA method, and very low-expressed transcripts were excluded from the analysis. Gene expression profiles of exposed samples and controls were compared to select differentially expressed transcripts (absolute fold-change ≥2 and corrected *p*-values ≤0.05). Statistical analysis was performed using Oneway ANOVA adjusted for multiple comparison by the Benjamini-Hochberg method. Microarray data are available in the ArrayExpress database (www.ebi.ac.uk/arrayexpress) under accession numbers E-MTAB-4458.

Data were analyzed by QIAGEN’s Ingenuity^®^ Pathway Analysis (IPA^®^ QIAGEN Redwood City, www.qiagen.com/ingenuity).

### RNA Extraction and qRT-PCR

RNA was extracted using Trizol reagent (Invitrogen). For qRT-PCR, 1 μg total RNA was reverse-transcribed (QuantiTect Reverse Transcription Kit, QIAGEN), according to the manufacturer’s instructions. qRT-PCR experiments were conducted using Applied Biosystem 7300 Real-Time PCR System and Power SYBR Green Master Mix (Applied Biosystems). The subsequent analysis was performed according to the model proposed by Hellemans et al. for qRT-PCR relative quantification and inter-run calibration with proper error. Data were normalized to the relative expression of three reference genes (*Actin*, *Tubulin* and *Beta-2 microglobulin*) [23]. Primers were designed using NCBI Primer Blast tool (http://www.ncbi.nlm.nih.gov/tools/primer-blast) and are reported in S1 Table. Amplification efficiency was calculated from triplicate relative standard curves for each primer pair and primer sets with amplification efficiencies outside of the 85–115% range were discarded.

### Protein Extraction and Western Blotting

Total and nuclear proteins were prepared and quantified as already reported [15, 24]. Rabbit antibodies against p65 (Abcam, ab7970), p53 (Santa Cruz Biotechnology, sc-6243), c-Myc (Santa Cruz Biotechnology, sc-764), p-Akt S473 (Cell Signaling, 3787), and topoisomerase I (Abcam, ab109374), and mouse antibodies against β-actin (Sigma-Aldrich) and Akt1 (Cell Signaling, 2967) were used. Densitometry was performed with ImageJ software.

### Alkaline Comet Assay

Alkaline comet assay (CometAssay, Trevigen) was used to assess DNA damage after UV-C irradiation, as suggested by the manufacturer. Briefly, the cells were collected and suspended in cold PBS (1x10^5^ cells/ml). A total of 5x10^3^ cells were mixed with a pre-warmed solution of 1% low melting point agarose, pipetted onto comet slides and allowed to solidify at 4°C for 15 min. The slides were subsequently incubated in lysis solution (1 hr at 4°C, according to the manufacturer’s instructions), in alkaline unwinding solution (200 mM NaOH, 1 mM EDTA, pH >13) for 20 min at 4°C, and subjected to electrophoresis (40 min at 30 V and 300 mA) in 300 mM NaOH, 1 mM EDTA, pH >13. The slides were washed in 70% ethanol (5 min) and dried at 37°C. All steps were carried out in the dark. The slides were stained with SYBR Gold (1:10000, Invitrogen) for 15 min at 37°C. Images were acquired using a Zeiss Axioplan-2 microscope with a 40x objective. An average of 100 cells for each condition were randomly selected and acquired. Data from the comet assay were processed with Comet Assay IV software to determine the intensity of the comet tail (the percentage of DNA in the tail).

### TUNEL Assay

Apoptotic cells were visualized by TUNEL assay kit (In Situ Cell Death Detection Kit, Fluorescein, Roche), according to the manufacturer’s instructions, and counterstained with a 1 μg/ml DAPI solution. Cells were visualized under a Zeiss Axioplan-2 microscope. Analysis of the TUNEL assay was performed using ImageJ software. For each experimental point, about 1000 cells were checked and positive cells were counted. The percentage of apoptotic cells was calculated as the ratio between positive cells and total cells for each analyzed field.

### Statistical and Bioinformatics Analyses

Statistical analyses were performed using Student’s t-test. In all cases, probability *p-*values below 0.05 were considered significant. * and ** indicate *p*-value <0.05 and <0.01, respectively. Data from at least three independent experiments were considered for the statistical analysis. In particular we performed three independent treatments, using different batches of FRTL-5, and RNA preparations for the microarray analysis. New 3 independent treatments, conducted as reported above, were done for the microarray data validation by qRT-PCR or Western Blotting and for the time-dependence analyses. The same was done for the cell cycle analysis in UV-exposed cells. Comet and TUNEL assays were conducted on the same treated cells as well as the analyses of *p21* expression level by qRT-PCR. Fold change (FC) values were calculated as the ratio between average results in treated and control samples. The results are expressed as the mean ± standard deviation of three independent experiments.

The position of transcription factor (TF) binding sites in Tp53 promoter was identified by uploading its sequence ranging from -300/+150 bp to the Genomatix Software Suite (Genomatix Software GmbH, http://www.genomatix.de), and choosing a relative profile score of 80% [25].

## Results

### Low-Dose BPA Exposure Impairs the Transcriptome of FRTL-5 Cells in a Time-Dependent Manner

To characterize the direct effects exerted by BPA on thyrocytes, we applied a toxicogenomic approach on FRTL-5, a rat immortalized thyrocytes cell line. FRTL-5 cells display many differentiated functions (active iodide transport, thyroglobulin synthesis, etc) and they are considered a valuable model for studying thyroid cell transformation *in vitro* [26]. We have previously shown FRTL-5 sensitivity to environmental dose (10^−9^ M) of BPA assessing the expression of thyroid specific genes [15].

**Fig 1:**
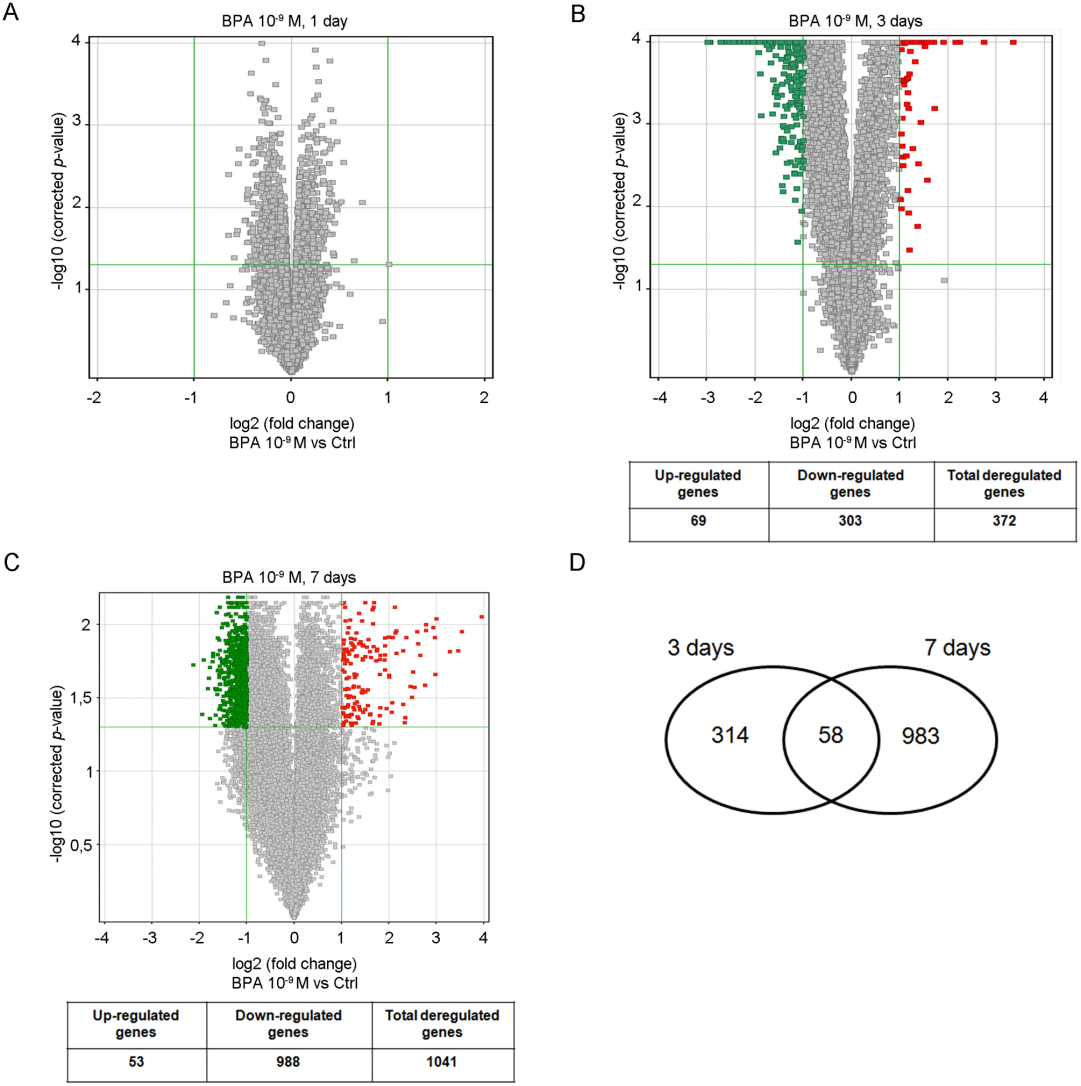
Time-dependent transcriptome perturba4tion induced by low-dose BPA in FRTL-5 cells. Volcano plots of microarray data after 1-day (**A**), 3-day (**B**) and 7-day (**C**) treatment with 10^−9^ M BPA compared to untreated cells. The *y*-axis value is the negative logarithm (base 10) of the corrected *p*-value. A green horizontal line on the plot represents the significant threshold for *p*-value. The *x*-axis is shown as the logarithm (base 2) of the FC in expression level between treated and control cells. The vertical green lines on the plot represent the thresholds for FC. Red dots are up-regulated probes; green dots are down-regulated probes. The number of down/up-regulated genes for each Volcano plot is reported in the underlying table. (**D**) Venn Diagram showing the gene set overlap between 3- and 7-day treatments.

To our aim, FRTL-5 cells were exposed for 1, 3, and 7-days to 10^−9^ M BPA, a dose within the range of BPA levels in human blood [2]. No major changes in the transcriptome were retrieved after 1-day treatment (FC ≥2, Fig 1A). Modifications in gene expression profiles were observed after 3- (Fig 1B) and 7-day (Fig 1C) treatments, with 372 and 1041 genes significantly deregulated in BPA-exposed cells, respectively. Most genes had a FC slightly greater than 2 at both time points (Fig 1B and 1C). The inconspicuous variation in FCs could be likely due to the low dose of BPA, as suggested by our previous results [15]. Only 31 genes were inhibited more than 4-fold at 3 days, and none at the later time. Similarly, 3 genes had a FC ≥4 both at 3- and 7-days. Only 58 genes (57 down- and 1 up-regulated) were similarly regulated at 3- and 7-days (Fig 1D), suggesting that the transcriptome alterations were quantitatively and qualitatively dependent on the duration of exposure.

We investigated the functional aspects of the deregulated set of genes by *in silico* functional annotation using a bioinformatics tool (IPA). This provided us with predictions of molecular networks, biofunctions, canonical pathways and upstream regulators altered in exposed FRTL-5 cells. Cell survival (decreased), cell death (increased), cell cycle (decreased), and cancer (increased), were among the most significant biofunctions predicted deregulated after 3-day exposure (S2 Table). IPA analysis of the 7-day data set highlighted the same biofunctions (S3 Table). Among the predicted “top 10” molecular networks, we found “DNA replication, recombination and repair” network at both 3- and 7-days, with different genes enriching the same network (S4 and S5 Tables, respectively). In Table 1, we report the time-dependent regulation of transcripts from the 7-day network (S1 Fig), as resulting from microarray experiments. The full analysis, including genes in the 3-day network, has been reported in S6 Table. The importance of “DNA replication, recombination and repair” network was strengthened also by the observation that the “checkpoint control” biofunction was predicted modulated at 3- and 7- days, although with different *p*-values (2.09E-08 and 1.21E-02). Genes involved in checkpoint control at 3 and 7 days, and their time-dependent regulation is reported in S7 Table. Noteworthy, Tp53 was predicted inhibited by the IPA analysis (S8 Table). This was expected considering its key role in regulating the biological functions discussed above.

**Table 1:** Genes enriched in the “DNA replication, recombination and repair” network modulated after 7-days BPA treatment.

Gene name	FC	*p*-value	FC	*p*-value
	3 days		7 days	
*Adsl*	-1.35	5.97E-03	**-2.36**	**3.26E-04**
*Ankfy1*	-1.04	7.67E-03	**-2.44**	**2.86E-03**
*Arpc3*	-1.43	8.57E-04	**-2.05**	**3.96E-05**
*Btbd1*	-1.10	1.74E-03	**-2.11**	**6.55E-04**
*Casp4*	2.42	1.76E-03	**-2.24**	**1.08E-04**
*Cops4*	-1.50	1.23E-03	**-2.13**	**1.15E-03**
*Cops6*	-1.23	1.08E-02	**-2.36**	**7.48E-03**
*Cops8*	-1.30	9.67E-03	**-2.39**	**1.61E-03**
*Cstb*	**-2.55**	**2.11E-03**	**-2.35**	**1.70E-03**
*Cul4a*	-1.06	9.33E-03	**-2.50**	**1.26E-04**
*Dcaf10*	1.08	1.08E-01	**-2.02**	**5.52E-03**
*Dcaf4*	-1.24	1.01E-01	**-2.30**	**1.79E-03**
*Dctn5*	**-2.04**	**4.98E-04**	**-2.35**	**1.12E-03**
*Dda1*	-1.64	9.95E-04	**-2.57**	**6.49E-04**
*Ddb1*	-1.14	1.04E-01	**-2.16**	**7.72E-03**
*Edaradd*	-1.35	1.28E-02	**-2.09**	**3.39E-03**
*Erap1*	-1.27	1.22E-02	**-2.09**	**2.21E-02**
*Fbxw4*	1.02	6.56E-01	**-2.06**	**2.62E-02**
*Fem1b*	1.51	3.54E-03	**-2.04**	**3.38E-02**
*Herpud1*	2.21	2.27E-05	**-2.33**	**2.03E-06**
*Hivep2*	1.12	1.15E-01	**-2.10**	**3.25E-03**
*Ibtk*	-1.06	8.79E-01	**-2.15**	**8.02E-03**
*Kdm2a*	-1.08	5.83E-02	**-2.00**	**6.47E-03**
*Klhl22*	1.01	7.08E-02	**-2.28**	**9.69E-03**
*Lta4h*	-1.55	1.86E-04	**-2.42**	**5.95E-04**
*Mdh1*	-1.88	7.13E-04	**-2.33**	**7.37E-04**
*Myo1e*	1.12	2.61E-01	**-2.07**	**1.95E-03**
*Nans*	**-2.11**	**6.11E-03**	**-3.16**	**2.45E-03**
*Pak1ip1*	**-2.23**	**1.37E-06**	**-2.01**	**5.69E-06**
*Psma6*	**-2.59**	**1.29E-04**	**-2.12**	**1.09E-04**
*RGD1310444*	**-2.52**	**6.83E-04**	**-2.95**	**5.63E-03**
*Scly*	-1.12	9.01E-01	**-2.34**	**7.25E-03**
*Taok2*	1.08	7.78E-02	**-2.29**	**3.65E-04**
*Trpc4ap*	-1.42	1.54E-02	**-2.64**	**2.67E-04**
*Uhmk1*	1.01	6.70E-02	**2.16**	**2.87E-02**
*Vprbp*	-1.07	7.74E-03	**-2.19**	**1.32E-03**
*Wdtc1*	1.39	4.53E-02	**-2.01**	**5.75E-03**
*Zmym4*	1.03	4.59E-01	**-2.06**	**2.77E-02**
*Zyg11b*	-1.08	6.91E-02	**-2.15**	**3.06E-03**

Overall, our data indicate that low-dose BPA exposure may modulate different biological functions such as cell death/proliferation, checkpoint control or DNA repair systems. Their impairment is conserved at different exposure times, although via the modulation of different gene sets. Indeed, the perturbed cellular functions and pathways are the same at different times, but the genes and the mechanisms involved in their impairment are flexible and adapted to the duration of exposure.

Genes enriched in the “DNA replication, recombination and repair” network at 7-day treatment are reported with relative FCs and *p*-values. FCs and *p-*values of the same genes at 3-day exposure are shown for comparison. Gene descriptions are listed in S7 Table. Significant FCs and *p*-values are in bold.

### Exposure to Low-Dose BPA Inhibits the Expression of Genes Involved in DNA Replication and Repair in a Time-Dependent Manner

**Fig 2:**
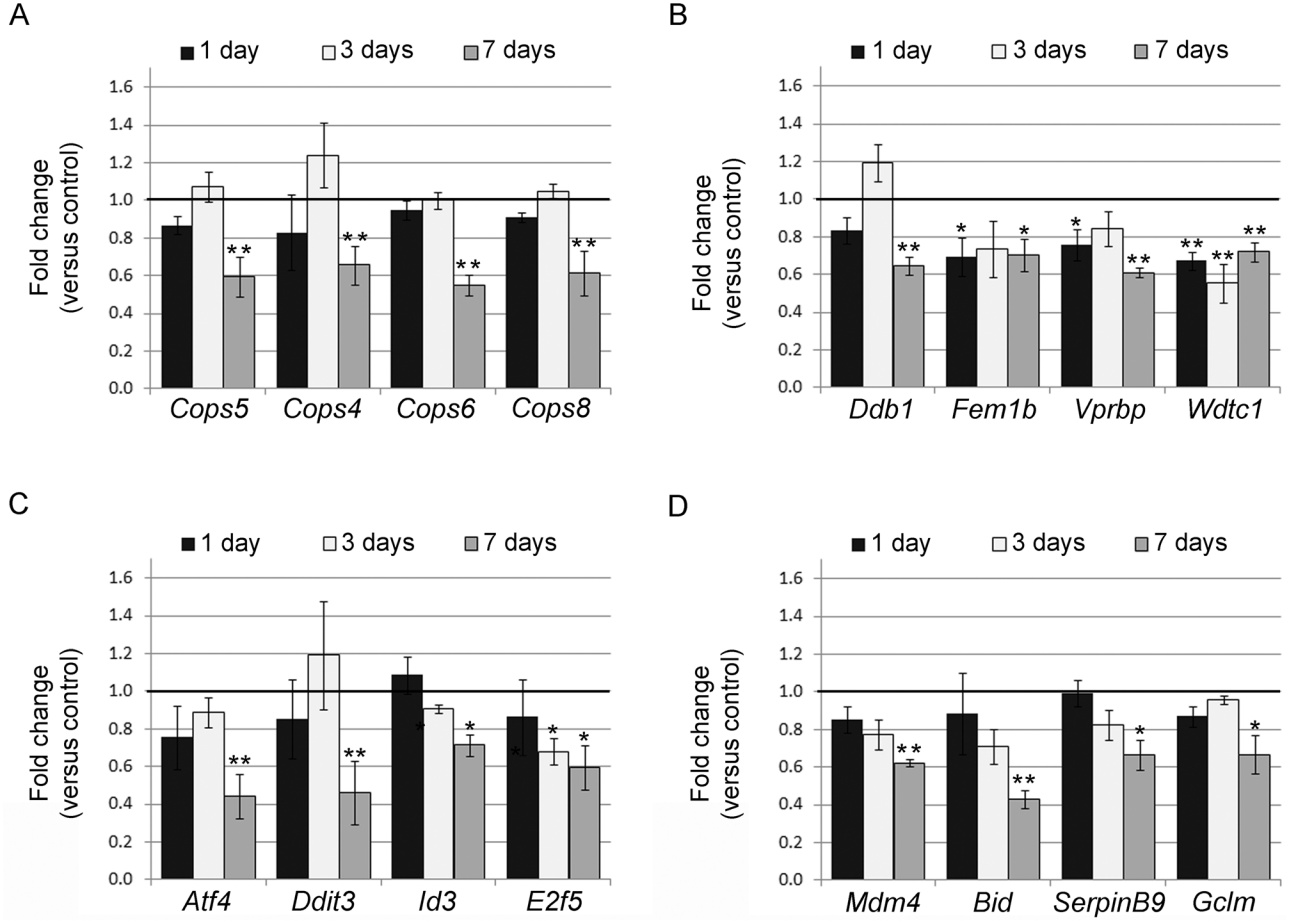
Time-dependent inhibition of genes in FRTL-5 cells treated with BPA verified by qRT-PCR. qRT-PCR analysis of some microarray down-regulated genes in FRTL-5 cells after 1-, 3-, and 7-day 10^−9^ M BPA treatment. In (**A**) and (**B**), genes enriched in the “DNA replication, recombination, and repair, developmental disorder, hereditary disorder” network, predicted in the 7-day gene set, are reported. In (**C**) and (**D**), transcripts leading to the prediction of p53 inhibition are shown. Data are reported as the ratio between mRNA content in BPA-treated and control samples. The mean ± standard deviation of three independent experiments is reported. *p-value <0.05; **p-value <0.01.

**Fig 3:**
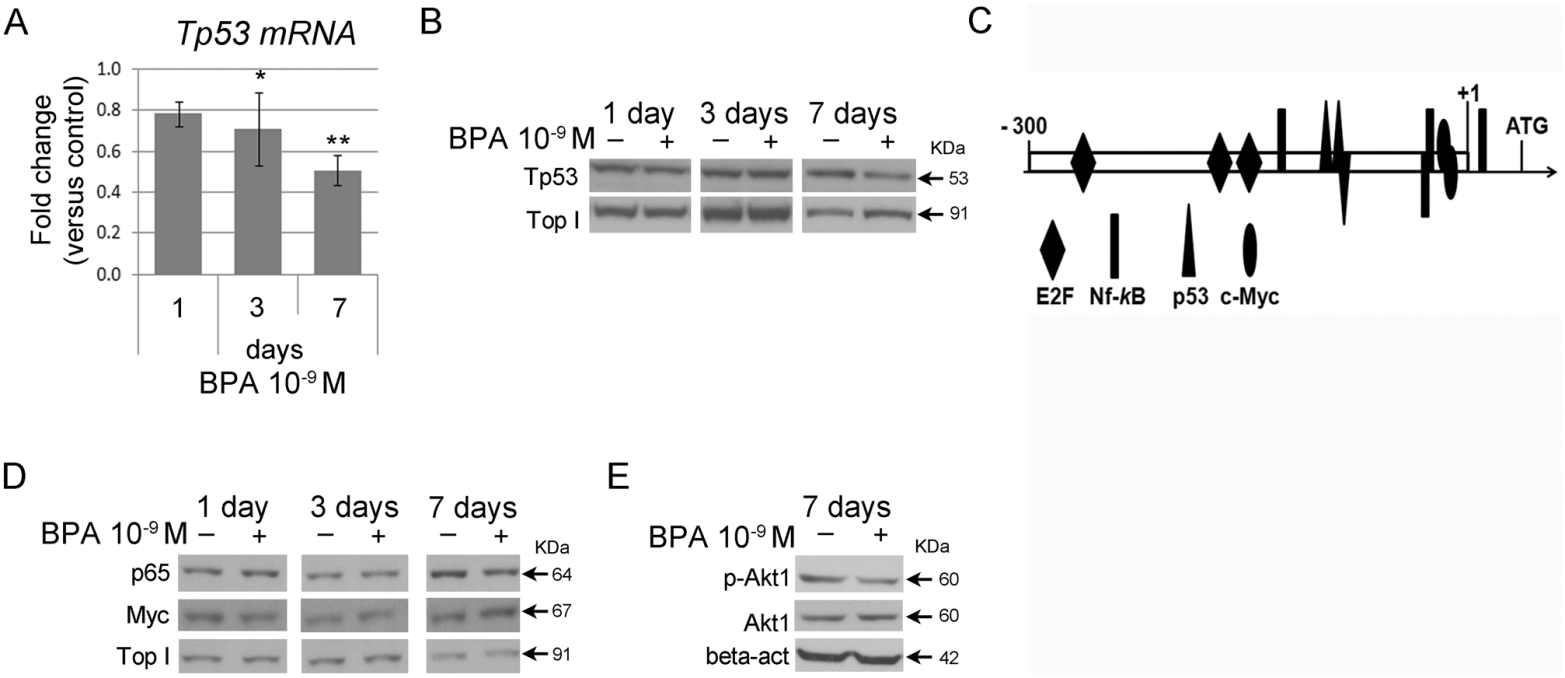
Tp53 and TF regulation following BPA treatment in FRTL5 cells. (**A**) qRT-PCR analysis of Tp53 transcript in FRTL-5 cells after 1-, 3-, and 7-day treatment with 10^−9^ M BPA. Data are reported as the ratio between transcript levels in BPA-treated and control samples. The mean ± standard deviation of three independent experiments is reported. *p-value <0.05; **p-value <0.01. (**B**) Western blot analysis of Tp53 nuclear protein levels in FRTL-5 cells after 1-, 3-, and 7-day treatment with 10^−9^ M BPA. (**C**) Schematic representation of *Tp53* promoter (-300/+130bp), depicting binding sites for TF predicted modulated by IPA. (**D**) Western blot analysis of p65 and c-Myc nuclear protein levels in FRTL-5 cells after 1-, 3-, and 7-day treatment with 10^−9^ M BPA. Topoisomerase I was used as loading control. Data are representative of three independent experiments (see S4 Fig). (**E**) Western blot analysis of p-Akt (Ser 473) and Akt in FRTL-5 cells after 7-day treatment with 10^−9^ M BPA. β-actin was used as loading control. Data are representative of three independent experiments (see S4 Fig).

Several reports described the direct genotoxic activity of high-dose BPA (μM range) as well as sensitization to DNA-damaging agents in different systems [27–33]. We used qRT-PCR analysis to validate the microarray data in FRTL-5 cells, independently treated with 10^−9^ M BPA for 7 days, and we focused on the transcripts involved in the above discussed functions (*Atf4*, *Bid*, *Ddit3*, *E2f5*, *Ddb1*, *Cops5*, *Cops4*, *Cops6*, *Cops8*, *Fem1b*, *Vprbp*, *Wdtc1*, *Mdm4*, *SerpinB9*, *Id3*, *Cat*, *Smad6*, *Gclm*, *Irf3*). qRT-PCR data showed good concordance with microarray data, although qRT-PCR FCs were generally lower (S9 Table). The cellular content of some of the above reported transcripts was assayed also at 1- and 3- days of exposure (Fig 2). In agreement with the microarray results, none of the assayed transcripts was inhibited at the shorter exposure times, with the exception of *E2f5*, one of the inhibited transcripts detected by microarray at 3 days (Fig 2C), and *Fem1b*, *Vprbp* and *Wdtc1* (Fig 2B). *Tp53* transcript was also included in this analysis: a slight inhibition of *Tp53* mRNA was detected after 3 days and became stronger at 7 days (Fig 3A). The nuclear levels of p53 protein were reduced only in cells exposed to 10^−9^ M BPA for 7 days (Fig 3B and S2 Fig).

To clarify the mechanism of BPA toxicity involved in the modification of the transcriptome at 7 days of exposure, we looked at the signalling pathways predicted deregulated by IPA analyses.

Firstly, we investigated the deregulation of mTOR pathway, predicted inhibited by IPA analysis of the 7-day data set, where the down-regulation of transcripts for TORC1 complex components, *mTOR* (FC = -2.18), *Rictor* (FC = -2.52) and *Mapkap1* (FC = -2.07), was retrieved. We assessed TORC1 inhibition verifying the phosphorylation of its target Akt on S473 that, as predicted, was reduced upon 7-day exposure (Fig 3E, S2 Fig).

Then, we focused on TFs predicted by the IPA analyses (S8 Table). Several of them have been described as positive or negative regulators of *Tp53* transcription: E2Fs (inhibited at 3 days) [34], Myc (inhibited) [35], p53 (inhibited) [36] and NF-*k*B (activated) [37]. We previously reported the action of NF-*k*B, involved in the network cited above, in FRTL-5 cells exposed to 10^−9^ M BPA for 1 day [15]. The arrangement of these TF binding sites in *Tp53* promoter sequence (-300/+130) is depicted in Fig 3C. As expected, we observed that p65, a negative regulator of *Tp53*, is accumulated in the nucleus after 1-day exposure (Fig 3D, S2 Fig) concomitantly with the peak in oxidative stress, tested by determining ROS content (S3 Fig). At the same time point, we detected a reduction in c-Myc, a positive regulator of *Tp53* promoter (Fig 3D, S2 Fig).

These findings suggest a sequence of events leading to the inhibition of *Tp53* mRNA, initially attributable to the impaired activity of ROS-responsive TFs such as NF-*k*B, and subsequently to the inhibition of E2Fs and of p53 itself, all positive regulators.

### Long-Term Exposure to Low-Dose BPA Exacerbates the Inhibition of Genes Involved in the DNA Replication and Repair Network

**Fig 4:**
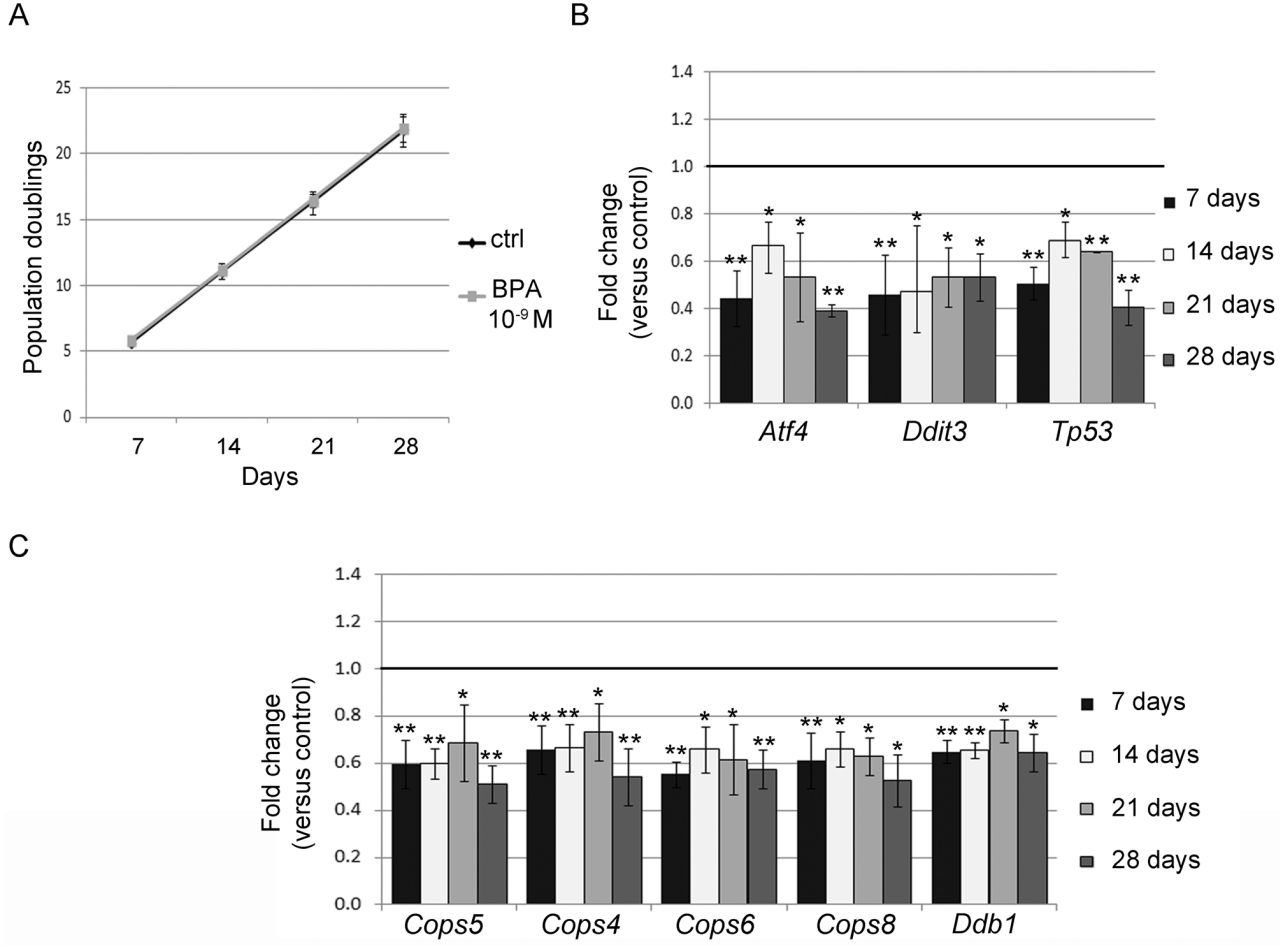
Transcript inhibition in long-term BPA-exposed FRTL-5 cells. (**A**) Proliferation rate analysis of FRTL-5 cells exposed for 28 days to 10^−9^ M BPA. Cells were counted every 7 days and the population doubling calculated as described in Material and Methods. Data are reported as mean ± standard deviation of three independent experiments. (**B**) qRT-PCR analysis of *Atf4*, *Ddit3*, *Tp53* and (**C**) of *Cops4*, *Cops5*, *Cops6*, *Cops8* and *Ddb1* in FRTL-5 cells treated for 28 days. Data are reported as the ratio between mRNA levels in 10^−9^ M BPA-treated and control samples. The mean ± standard deviation of three independent experiments is reported. *p-value <0.05; **p-value <0.01.

Living organisms are continually exposed to nanomolar (nM) doses of BPA. To mimic this condition, we assessed the effects of long-term exposure in FRTL-5 cells continuously maintained in BPA for 28 days. As BPA impairment of cell proliferation and death was previously described in other cellular systems [38, 39] and predicted by our IPA results, we monitored cell growth weekly. We did not observe any statistically significant difference in proliferation rate (population doubling) between control and 10^−9^ M BPA-treated cells (Fig 4A). No major changes in cell death and cell distribution in the different phases of cell cycle were revealed by cytofluorimetric analysis (S4 Fig).

Further, we tested the levels of some transcripts belonging to the “DNA repair” network (S1 Fig), in the long-term exposure conditions. We pointed our analysis on components of the COP9 signalosome, a protein complex involved in DNA damage response (DDR) and nucleotide excision repair (NER) [40], including *Cops4*, *Cops6*, *Cops5* and *Cops8* (Fig 4B). *Ddb1* was also selected (Fig 4B) for its involvement in recognizing UV-induced DNA lesions in the NER pathway [41]. In addition, we monitored the levels of *Atf4*, *Ddit3*, and *Tp53* mRNA, key players in DDR (Fig 4C). To this aim, FRTL5 cells were exposed to 10^−9^ M BPA for 28 days, and RNA prepared weekly. As expected, all the genes were inhibited after 7-day exposure to 10^−9^ M BPA. At longer exposure times, some of them retained the level of inhibition previously reached while some others were further inhibited (Fig 4B and 4C).

The reported findings suggest that exposure longer than 7 days stabilizes/reinforces the effects of BPA on “DNA repair” network transcripts. This effect was quantitative, in terms of fold change, rather than qualitative, as the same transcripts were found at the different time points, unlike the results obtained at shorter exposures (3 days versus 7 days).

### BPA Reduces the Ability of FRTL-5 Cells to Promptly Recover following DNA Damage

**Fig 5:**
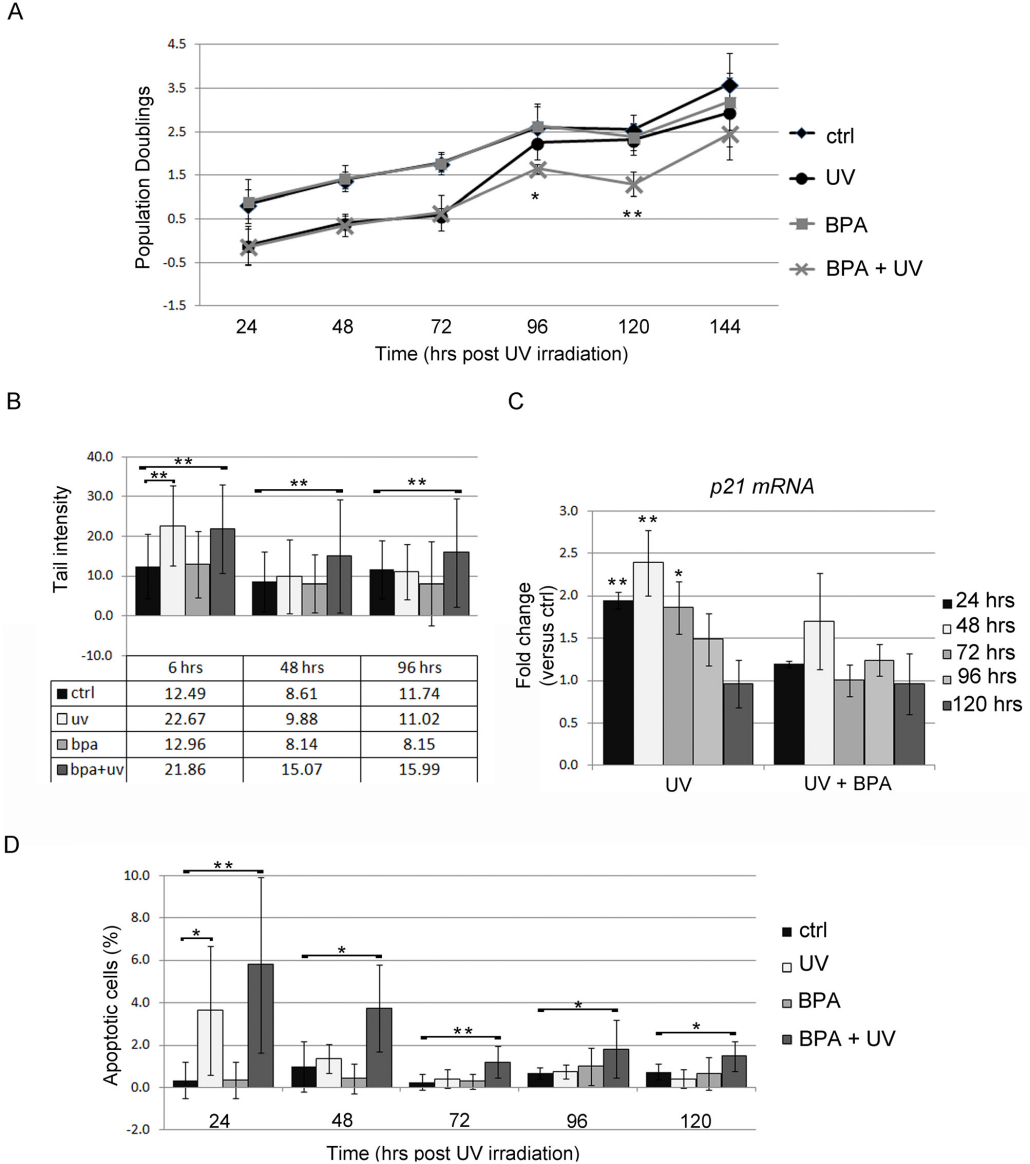
Analysis of FRTL-5 cell response to UV-C irradiation following long-term BPA treatment. (**A**) Proliferation rate analysis of FRTL-5 cells treated for 28 days with 10^−9^ M BPA and then subjected to UV-C irradiation. Cells were counted every 24 hrs until 144 hrs post-irradiation. Population doubling was calculated as described in Materials and Methods. Data are reported as mean ± standard deviation of three independent experiments. (**B**) Quantification of DNA damage by comet assay. Data are reported as mean ± standard deviation of the tail intensity of around 100 cells analyzed for each point. (**C**) qRT-PCR analysis of the pattern of *p21* transcript levels following UV-C irradiation. Data are reported as the ratio between *p21* transcript levels in irradiated and control cells. The mean ± standard deviation of three independent experiments is reported. *p-value <0.05; **p-value <0.01. (**D**) Quantification of apoptotic cells by TUNEL staining. Data are reported as percentage of TUNEL positive cells per total cell number identified by DAPI staining. The results are expressed as mean ± standard deviation of several fields analyzed in three independent experiments.

The above findings indicate that long-term exposure to BPA stabilizes the inhibition of transcripts encoding proteins involved in DNA repair. We wondered if the BPA-induced deregulations of these genes could impair the ability of FRTL-5 cells to proper react to DNA damage inducers. It was reported that NER-deficient Chinese hamster ovary cells were sensitized to apoptosis after UV-C exposure [42] through a mechanism requiring cell replication. We therefore investigated the response to UV-C of FRTL-5 cells, irradiated after treatment for 28 days with BPA. The response of BPA-exposed cells to UV-C irradiation was tested by assaying cell proliferation/death and DNA damage level. Cell proliferation rate was monitored every 24 hrs for 5 days after irradiation by calculating population doublings. No changes between BPA-treated and control cells were retrieved at any time (Fig 5A). In UV-C irradiated cells, population doubling was similar in absence or presence of BPA up to 72 hrs after irradiation. At 96 hrs and 120 hrs following UV-C irradiation, BPA-treated cells showed significantly lower population doublings (Fig 5A) than untreated cells, suggesting their need of a longer time to recover from the damage. To directly test the ability of the cells to repair the UV-C-induced DNA damages, we performed a comet assay measuring tail intensity at different times following irradiation (Fig 5B) in the above described experimental setting. The content in damaged DNA was similar in UV-irradiated cells, in presence or absence of BPA, when determined 6 hrs post-irradiation (Fig 5B). Noteworthy, no differences between control and BPA exposed cells were detected, indicating that BPA (10^−9^ M) had no direct genotoxic effect in FRTL-5 cells. Later on after irradiation, at 48 and 96 hrs, the untreated UV-irradiated cells, presented tail intensity similar to controls. At the same time points, irradiated BPA-treated cells still showed a significantly higher content of damaged DNA than control cells, indicating an impaired ability of BPA exposed cells to promptly repair the DNA lesions.

These data indicate an inadequate response to DNA damage, as predicted by IPA analyses. Tp53 is known to increase *p21* transcript expression upon UV-C irradiation, inducing a block in cell cycle progression [43]. We analysed the level of *p21* transcript in the samples. As expected, *p21* transcript accumulated in UV-C irradiated cells at 24 hrs and 48 hrs, increasing slowly and steadily and then decreasing in the same manner back to control levels. After UV-C irradiation, this pattern of p21 accumulation and recovery was not observed in BPA-treated cells (Fig 5C), in agreement with the phenotypic result reported above. We checked also for the apoptosis after irradiation via TUNEL assay (Fig 5D) and FACS analysis (data not shown). Both the assays revealed that at 24 hrs post irradiation the apoptotic levels are comparable for BPA-treated and the untreated cells. At later time points (48, 72, 96 and 120 hrs), the untreated cells, that promptly repaired the induced damage, had apoptosis at the basal level. On the other side, the BPA treated cells, being slower in the repairing process, had significantly higher apoptosis levels until 120 hrs post irradiation.

In summary, BPA-exposed cells were unable to efficiently repair DNA damage after UV-C irradiation due to impairment of the COP9 signalosome and in the p53-p21 action. Taken together, our findings suggest that long-term exposure to BPA does not exert a direct genotoxic activity, but impairs cellular response to genotoxic insults by acting on machinery involved in DNA repair.

## Discussion

BPA activity as a “thyroid disruptor” has been proposed in several studies [9–12, 14], but remains controversial. Thyroid is highly susceptible to environmental pollutants, which may act as either genotoxic or non-genotoxic carcinogens. For example, polybrominated diphenyl ethers (PBDEs) may induce abnormal thyroid cell proliferation, favouring a precancerous state [44]. Very few data are available on the molecular mechanisms of BPA toxicity related to thyroid dysfunctions and carcinogenesis [45]. To fulfil this gap we applied a toxicogenomic approach in the analyses of BPA effects in immortalized thyrocytes. We show, for the first time, that low-dose BPA is able to lower the efficiency of DNA repair systems, an effect that could indirectly contribute to carcinogenesis.

In particular, we report a time-dependent modification of the FRTL-5 transcriptome following low-dose BPA exposure. We observed, over the time, an increment in the number of deregulated genes and different gene sets at the analysed time points. However, we showed that the same cellular processes (e.g. cell death, proliferation) are impaired at different exposure times. This suggests that impairment of pathways and biological functions is more “stable” than the impairment of single genes. Thus, phenotype anchoring should focus on the former rather than the latter, as the analysis of changes in expression profiles of individual genes has rarely proven to be of use.

No relevant transcriptomic changes were detected at short exposure times pointing out the importance of exposure duration in evaluating the effects of environmental/low-dose BPA exposure. Furthermore, this suggests that time is required to activate signalling pathways beyond the threshold level if the signal is slight, unlike estrogen receptors. Time is also required to induce significant transcriptomic changesthat will be slight if the strength of the signal is low, but significant in terms of the number of involved genes. Looking at our data, we can speculate that although the conventional analysis of significantly deregulated transcripts (the so-called top genes) is informative and more straightforward, it does not necessarily identify the actual mechanisms of toxicity, highlighting the importance of considering slightly regulated genes.

**Fig 6:**
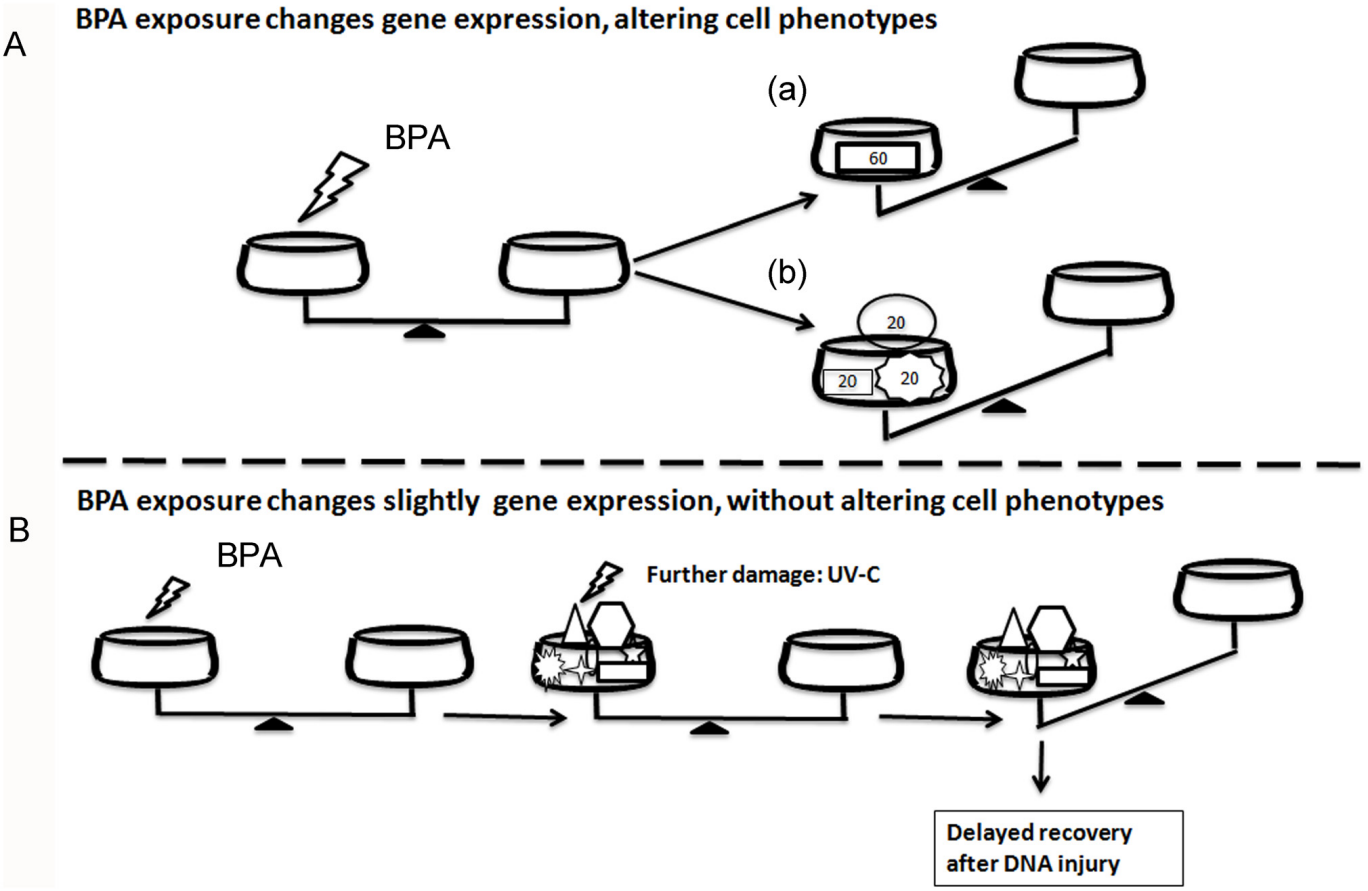
Summary of BPA mechanisms of toxicity. (**A**) Cells exposure to high-dose of BPA can strongly deregulates the expression of a single (a) or few genes in the same pathway (b), impairing a cellular function. (**B**) Low-dose BPA exposure can induce a slight deregulation of many genes in the same pathway without compromising a specific phenotype. If a second injury is applied, the damage is highlighted by phenotypic changes potentially representing a hazard for health.

Overall, our findings suggest that the slight perturbation of many transcripts involved in the same functions can lead to a severe impairment of cellular activity (Fig 6B), similar to that produced by a robust alteration of one or few master genes (Fig 6A).

To our knowledge, the above hypotheses have been put forward since the emergence of toxicogenomics, but not clearly highlighted in the majority of published studies, suggesting the need for a different approach to analysing toxicogenomic data in chemical/drug testing.

Again, our results support the idea that continuous and low-level exposure to BPA, resembling the environmental exposure condition, is unable to exert visible damage on thyroid cells but can induce a slight deregulation of many genes. Thus, cells will be predisposed to develop an inadequate response to further stressors (Fig 6B), as shown here in the case of machinery controlling DNA damage *(Tp53*, *Ddit3*, *Ddb1*, and components of the COP9 signalosome). Several reports described the DNA-damaging activity of BPA at high doses (μM range) [27–30, 33]. Our results, indicating that BPA (nM range) is able to impair the correction of DNA damage, are in agreement with the previous finding that exposure to BPA enhances sensitization to DNA-damaging agents [32]. Low-dose and long-term BPA exposure induces only a slight deregulation of many genes involved in cell proliferation/death, cancer, and DNA repair, allowing cell adaptation to the insult and proliferation. This effect is achieved by weakening control systems such as the p53-p21 axis and COP9 signalosome. No major phenotypic changes are observed until a further damaging event occurs (Fig 6B). Only at this point the cell damage is manifested through an inadequate cell response leading to the death or possible proliferation of cells carrying mutations. Thus, BPA is able to “indirectly” exert a genotoxic activity by impairing DDR. Thyroid cells are constantly threatened by DNA damage due to both exogenous and endogenous stressors [16]. The weakening of response to DNA damage may be the mechanism by which BPA is able to increase the rate of mutations when a second stressor is applied. Similar mechanisms were described in a rat study showing that perinatal exposure to low doses of BPA increases carcinogenic susceptibility to a chemical administrated in mammary gland later in life [46].

The described mechanism of low-dose BPA toxicity may help to explain the divergence between the increased incidence of thyroid dysfunction/cancer, attributable to environmental pollutions, and the scarcity of supporting experimental data. Only one report investigated the role of BPA in a two-stage thyroid carcinogenesis using it as a second stressor [45]. In conclusion, the idea that xenobiotics can pose a hazard for human health by lowering the response to further injury may be extended to environmental monitoring of other chemicals or stressors. Overall, the described results are in agreement with our previous report showing that exposure to low dose BPA impaired pancreatic islets activity in a similar manner [47].

Further studies are required to evaluate the role of BPA in thyroid carcinogenesis. However, our data suggest the need for a more precise understanding of the carcinogenic activity of BPA as well as other endocrine-disrupting chemicals, considering that “the real environment” means not only low-doses but also exposure to several potentially damaging agents, whose combination can result in worse effect.

## Conclusions

In this report, we show that the exposure to environmental dose of BPA significantly impairs the thyrocytes transcriptome in a time dependent manner. The effects were slight but involving many genes grouped in specific pathways. Thus, low-dose BPA exposure results in a stockpile of slight impairments determining phenotypic changes as here shown for genes with relevant functions in DNA damage recognition and repair. Indeed, the retrieved changes are not strong enough to compromise cell function in a steady condition, but impair the ability of the cell to properly react to a further stressor like a DNA damaging agent.

## Supporting Information

S1 Fig “DNA replication, recombination, and repair, developmental disorder, hereditary disorder” network highlighted at 7-day exposure. (TIF)

S2 Fig Relative protein levels in BPA-treated FRTL-5 cells. Relative p53 (**A**), p65 (**B**), c-Myc (**C**) nuclear protein levels in FRTL-5 cells after 1-, 3-, and 7-day treatment with 10^−9^ M BPA were quantified by Western blotting and normalized to Topoisomerase 1 levels. (**D**) Relative p-AKT and AKT in total protein extracts in FRTL-5 cells after 7-day treatment with 10^−9^ M BPA were quantified by Western blotting and normalized to β-actin levels. Densitometry was performed with ImageJ software. Data are reported as the ratio between BPA-treated and control samples. The mean ± standard deviation of 3 independent experiments is reported. *p-value <0.05; **p-value <0.01.
(TIF)

S3 Fig Time-dependent oxidative stress induced by BPA in FRTL-5 cells. Cellular ROS levels after 1-, 3-, and 7-day treatment with 10^−9^ M BPA are shown. Data are reported as the ratio between BPA-treated and control samples. The mean ± standard deviation of 3 independent experiments is reported. *p-value <0.05; **p-value <0.01.
(TIF)

S4 Fig Cell cycle and cell death analysis of long-term BPA-treated FRTL-5 cells. Cells were continuously treated with 10^−9^ M BPA for 28 days. Every 7 days a fraction of cells was re-plated and another fraction used for FACS analysis.
(TIF)

S1 Table Primer sequences used for qRT-PCR. For each gene analysed by qRT-PCR the gene description, the forward and reverse primers used are reported.
(DOCX)

S2 Table IPA biofunctions deregulated following 3-day BPA treatment in FRTL-5 cells. IPA biofunctions with a significant activation state prediction are reported. IPA *z*-score predicts the effect of gene expression changes on significantly enriched biological functions. The activation state of a function is predicted increased for *z*-score ≥2 and decreased for *z*-score ≤-2.
(DOCX)

S3 Table IPA biofunctions deregulated following 7-day BPA treatment in FRTL-5 cells. IPA biofunctions with a significant activation state prediction are reported. IPA *z*-score predicts the effect of gene expression changes on significantly enriched biological functions. The activation state of a function is predicted increased for *z*-score ≥2 and decreased for *z*-score ≤-2.
(DOCX)

S4 Table Top 10 IPA associated functional networks modulated by 3-day BPA treatment in FRTL-5 cells. The 10 higher scored functional networks are listed, with the relative score and the number of molecules belonging to the network.
(DOCX)

S5 Table Top 10 IPA associated functional networks modulated by 7-day BPA treatment in FRTL-5 cells. The 10 higher scored functional networks are listed, with the relative score and the number of molecules belonging to the network.
(DOCX)

S6 Table Genes in the “DNA replication, recombination and repair” network predicted deregulated after 3- and 7-day exposure to BPA. The union of genes enriched in the “DNA replication, recombination and repair” network at 3 and 7 days is reported with respective FCs and *p*-values. Significant FCs and *p*-values are in bold.
(DOCX)

S7 Table Genes in the “checkpoint control” category predicted deregulated after 3- and 7-day exposure to BPA. The union of genes enriched in the “checkpoint control” category at 3 and 7 days are reported with respective FCs and *p*-values. Significant FCs and *p*-values are in bold.
(DOCX)

S8 Table Top IPA predicted transcription regulators after 7-day treatment with 10^−9^ M BPA in FRTL-5 cells. IPA overlap *p-*values (Fisher's exact test) are used to assess whether there is a statistically significant overlap between genes in the data set and genes regulated by a specific transcriptional regulator. The IPA *z*-score predicts the effect of gene expression changes on significantly predicted transcriptional regulators. The activation state of a transcriptional regulator is predicted activated for *z*-score ≥2 and inhibited for *z*-score ≤-2.
(DOCX)

S9 Table Microarray validation of genes deregulated in the 7-day data set by qRT-PCR. Genes whose deregulation has been verified by qRT-PCR are listed. For each of them, the gene description, the fold change and relative *p*-value of both qRT-PCR and microarray experiments are reported.
(DOCX)

## Abbreviations

BPA: Bisphenol A
TH: thyroid hormone
TSH: thyrotropin
FRTL-5: rat follicular thyroid cell line 5
IPA: Ingenuity Pathway Analysis
TF: transcription factors
FC: fold change
DDR: DNA damage response
NER: nucleotide excision repair
