# Supplementary figures and images for: “Stockpile” of Slight Transcriptomic Changes Determines the Indirect Genotoxicity of Low-Dose BPA in Thyroid Cells
Source: PLoS One. 2016 Mar 16;11(3):e0151618. doi: 10.1371/journal.pone.0151618 (PMC4794173; doi:10.1371/journal.pone.0151618)

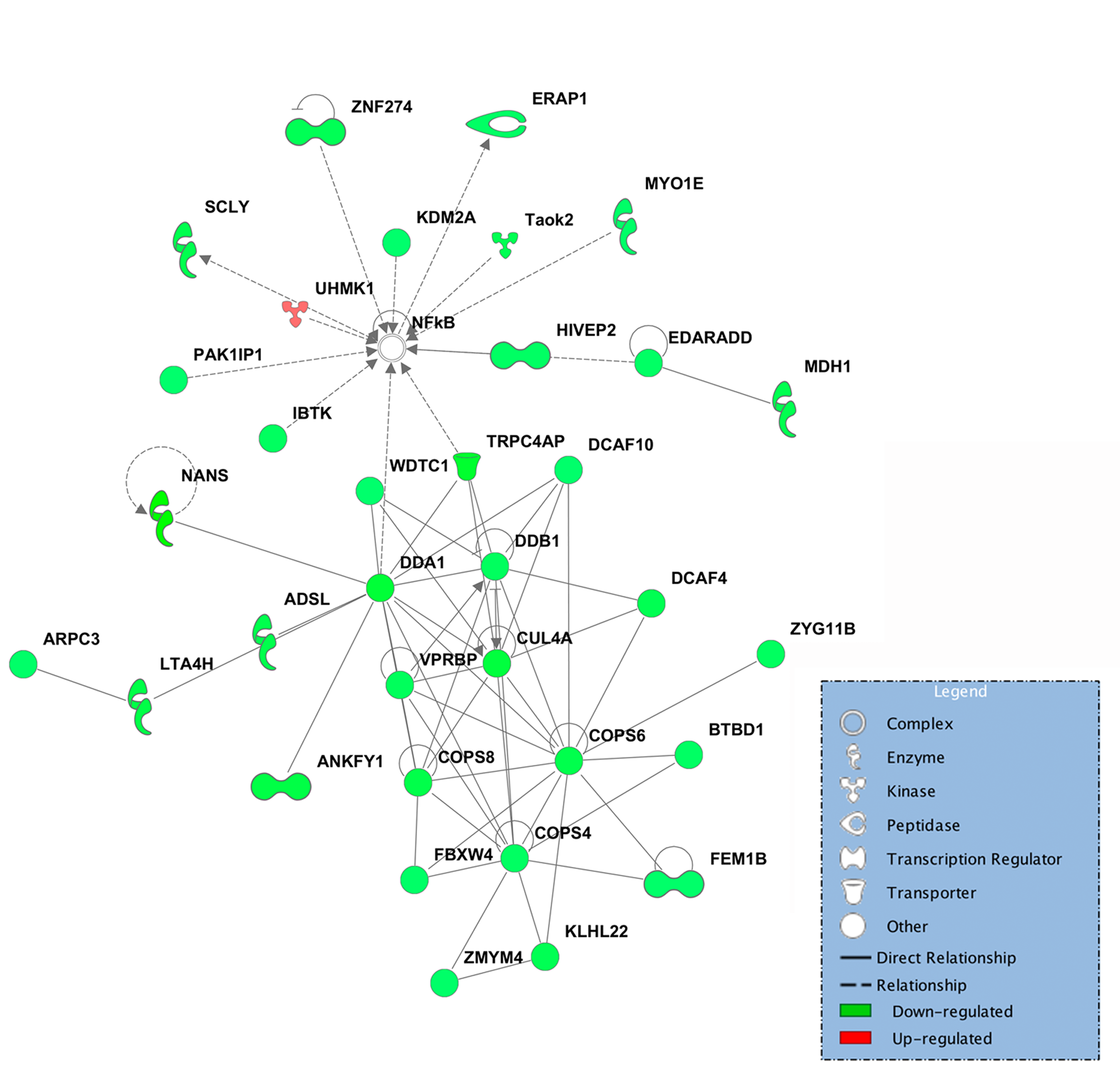

Supplement: S1 Fig — (TIF) [file pone.0151618.s001.tif]

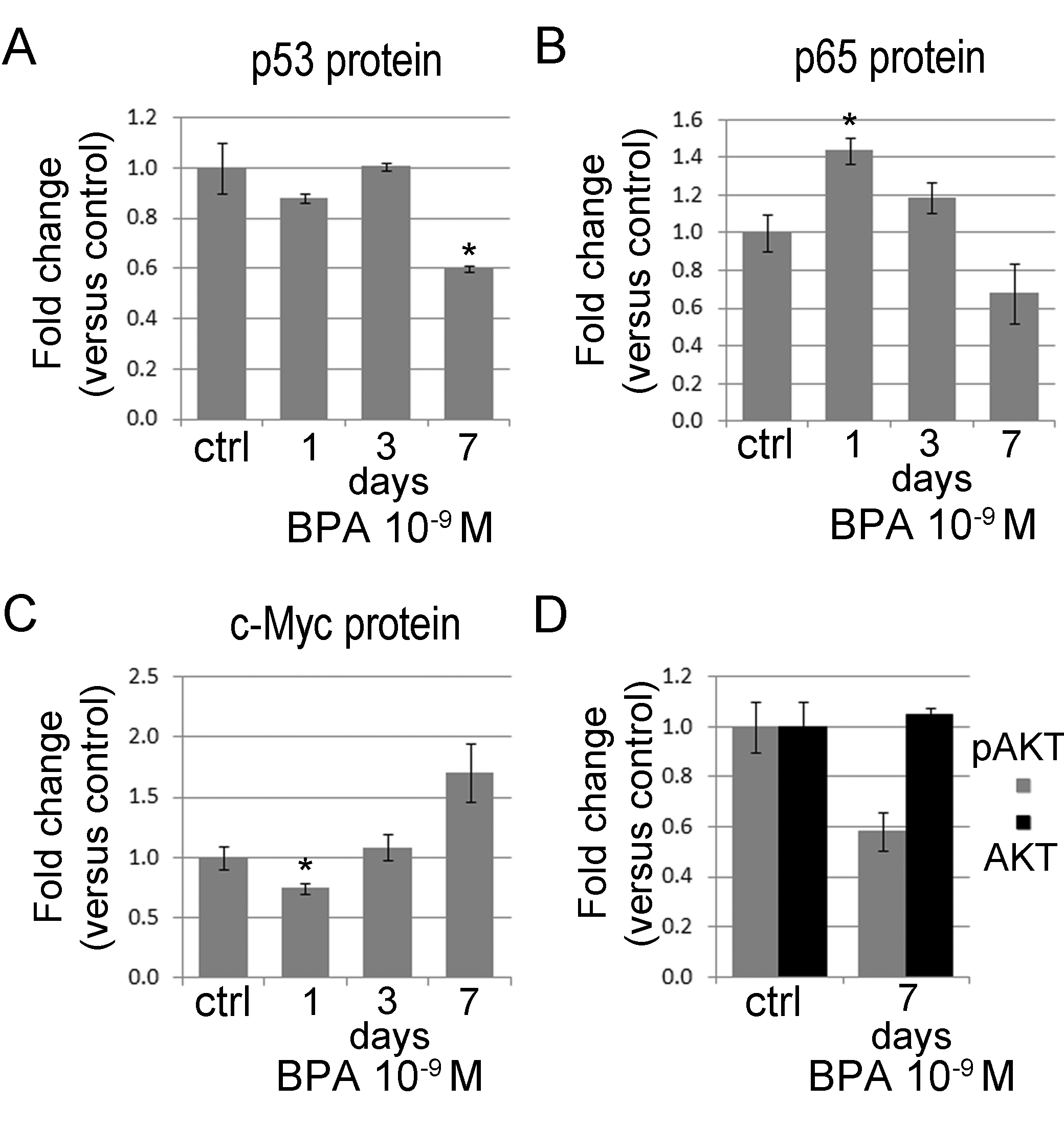

Supplement: S2 Fig — Relative p53 (A), p65 (B), c-Myc (C) nuclear protein levels in FRTL-5 cells after 1-, 3-, and 7-day treatment with 10−9 M BPA were quantified by Western blotting and normalized to Topoisomerase 1 levels. (D) Relative p-AKT and AKT in total protein extracts in FRTL-5 cells after 7-day treatment with 10−9 M BPA were quantified by Western blotting and normalized to β-actin levels. Densitometry was performed with ImageJ software. Data are reported as the ratio between BPA-treated and control samples. The mean ± standard deviation of 3 independent experiments is reported. *p-value <0.05; **p-value <0.01. (TIF) [file pone.0151618.s002.tif]

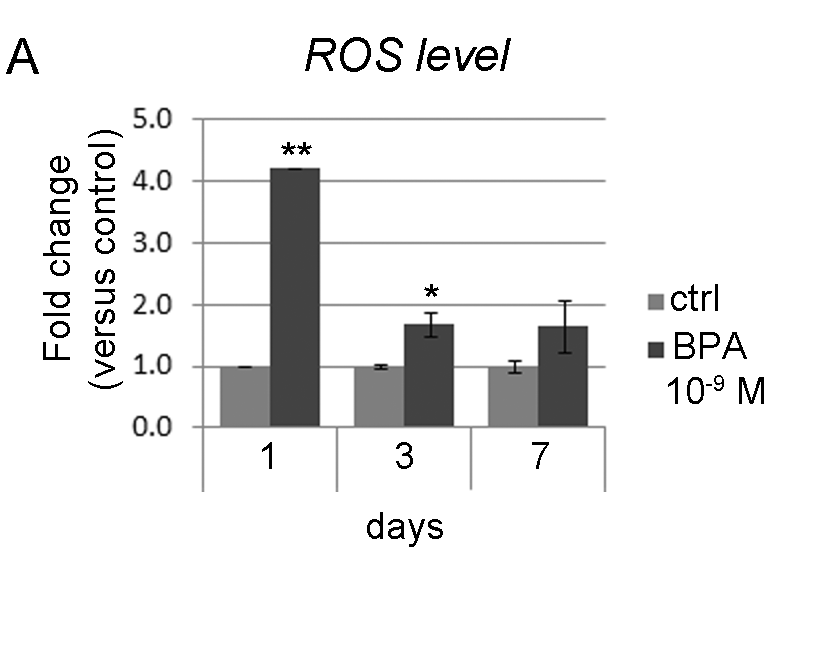

Supplement: S3 Fig — Cellular ROS levels after 1-, 3-, and 7-day treatment with 10−9 M BPA are shown. Data are reported as the ratio between BPA-treated and control samples. The mean ± standard deviation of 3 independent experiments is reported. *p-value <0.05; **p-value <0.01. (TIF) [file pone.0151618.s003.tif]

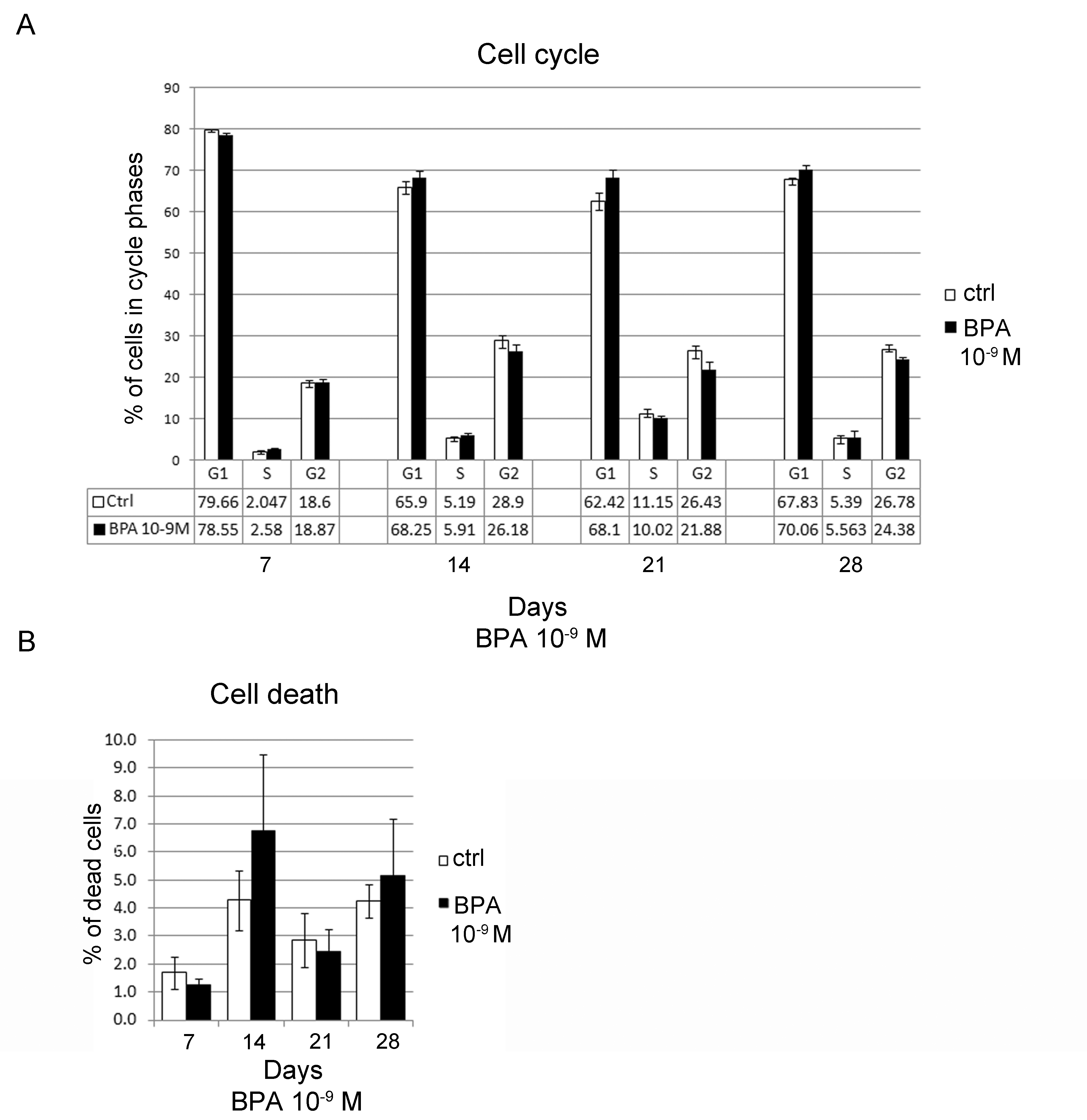

Supplement: S4 Fig — Cells were continuously treated with 10−9 M BPA for 28 days. Every 7 days a fraction of cells was re-plated and another fraction used for FACS analysis. (TIF) [file pone.0151618.s004.tif]
