# Supplementary material for: “Stockpile” of Slight Transcriptomic Changes Determines the Indirect Genotoxicity of Low-Dose BPA in Thyroid Cells
Source: PLoS One. 2016 Mar 16;11(3):e0151618. doi: 10.1371/journal.pone.0151618 (PMC4794173; doi:10.1371/journal.pone.0151618)
Supplement: S1 Table — For each gene analysed by qRT-PCR the gene description, the forward and reverse primers used are reported. (DOCX) [file pone.0151618.s005.docx]

**S1 Table.** Primer sequences used for qRT-PCR

| Gene name | Gene description | Forward primer 5’-3’ | Reverse primer 5’-3’ |
| --- | --- | --- | --- |
| *Atf4* | activating transcription factor 4 | CCAGTCGGGTTTGGGGGCTG | AAGGCATCCTCCTTGCCGGTG |
| *Bid* | BH3-interacting domain death agonist | CCAGGCATCTCGCCCAAGCA | GCGGAAGACGTCACGGAGCA |
| *Cat* | catalase | CACTCAGGTGCGGACATTCT | CAGGGTGGACGTCAGTGAAA |
| *Cops5* | COP9 constitutive photomorphogenic homolog subunit 5 | GGCGGCGAAACCCTGGACTA | ACTCGTATGCAGCAGCCTGGG |
| *Cops4* | COP9 constitutive photomorphogenic homolog subunit 4 | GGCTGCCGTGCGACAGGATT | AGGCTGACGTTCTCGTTCACCA |
| *Cops6* | COP9 constitutive photomorphogenic homolog subunit 6 | GCGGCCTATGCAGGTGATTGGG | TGGACGTGGATGTCTGAGGGGT |
| *Cops8* | COP9 constitutive photomorphogenic homolog subunit 8 | GCCTTTGCCCTGGTCTCGCA | GCAAGCTGCTGCTCGTTGGG |
| *Ddb1* | damage-specific DNA binding protein 1 | GGCGGAGCCCAAACAGGGTC | CGCATGAGGTCGCCCACCAG |
| *Ddit3* | DNA-damage inducible transcript 3 | AAGATGAGCGGGTGGCAGCG | CCGGTTTCTGCTTTCAGGTGTGGT |
| *E2f5* | E2F transcription factor 5 | CGGCGTCCTGGATCTCAAAGCG | CCAGCACCTACACCCTTCCACT |
| *Fem1b* | fem-1 homolog b | CTCCACACCCCTCATCATCG | ACCGTCAATGACATACCCGT |
| *Gclm* | glutamate-cysteine ligase, modifier subunit | GAAAAAGTGTCCGTCCACGC | CATCTGGAAACTCCCTGACCA |
| *Id3* | inhibitor of DNA binding 3 | TGGACGACATGAACCACTGC | CAGCTGTCTGGATCGGGAG |
| *Irf3* | interferon regulatory factor 3 | GCACTGTATGCGCAGGCTGGA | GCACCACTGGCTTCCGCCC |
| *Mdm4* | MDM4, p53 regulator | TCAAGACCGACTGAAGCACG | TAGAAACCACCAAGGCAGGC |
| *p21* | cyclin-dependent kinase inhibitor 1A | ATCCAGACATTCAGAGCCACAG | ACGAAGTCAAAGTTCCACCGT |
| *SerpinB9* | serine (or cysteine) peptidase inhibitor, clade B, member 9 | GCATCAAGGCTGCAAATGCT | TGTTCAAACCAAGTGCCTGAG |
| *Smad6* | SMAD family member 6 | CAAGCCACTGGATCTGTCCG | GCCCTGAGGTAGGTCGTAGA |
| *Tp53* | tumor protein p53 | AACCGCCGGCCCATCCTTAC | CTCGAAGCGCTCACGCCCAC |
| *Vprbp* | Vpr (HIV-1) binding protein | GGTGCCAAAAATCCAGCTCC | GCCACGCCCAAAATAATGCT |
| *Wdtc1* | WD and tetratricopeptide repeats 1 | ATACCTGGAGCGTGTGAAGC | CGGAGGGCATCATAGTGGTC |
