# Supplementary material for: “Stockpile” of Slight Transcriptomic Changes Determines the Indirect Genotoxicity of Low-Dose BPA in Thyroid Cells
Source: PLoS One. 2016 Mar 16;11(3):e0151618. doi: 10.1371/journal.pone.0151618 (PMC4794173; doi:10.1371/journal.pone.0151618)
Supplement: S2 Table — IPA biofunctions with a significant activation state prediction are reported. IPA z-score predicts the effect of gene expression changes on significantly enriched biological functions. The activation state of a function is predicted increased for z-score ≥2 and decreased for z-score ≤-2. (DOCX) [file pone.0151618.s006.docx]

**S2 Table.** IPA biofunctions deregulated following 3-day BPA treatment in FRTL-5 cells

| Categories | Diseases or Functions Annotation | *p*-Value | Predicted Activation State | Activation *z*-score | Number of molecules |  |
| --- | --- | --- | --- | --- | --- | --- |
| Cell Cycle | cell cycle progression | 1.68E-22 | Decreased | -2.133 | 80 |  |
| Cell Death and Survival | cell death of tumor cell lines | 3.21E-14 | Increased | 2.25 | 84 |  |
| Cell Cycle | M phase of tumor cell lines | 5.94E-13 | Decreased | -2.909 | 19 |  |
| Cell Death and Survival | cell death | 2.03E-11 | Increased | 2.515 | 133 |  |
| Cell Cycle | M phase of cervical cancer cell lines | 5.86E-11 | Decreased | -2.137 | 15 |  |
| Cell Death and Survival | necrosis | 1.96E-10 | Increased | 2.407 | 108 |  |
| Cell Death and Survival | apoptosis of cervical cancer cell lines | 1.44E-09 | Increased | 2.642 | 26 |  |
| Cellular Development, Cellular Growth and Proliferation | proliferation of tumor cell lines | 3.07E-09 | Decreased | -5.284 | 72 |  |
| Cellular Growth and Proliferation | proliferation of cells | 8.49E-09 | Decreased | -5.383 | 133 |  |
| Cell Cycle, DNA Replication, Recombination, and Repair | checkpoint control | 2.09E-08 | Decreased | -2.158 | 15 |  |
| DNA Replication, Recombination, and Repair | metabolism of DNA | 4.01E-08 | Decreased | -2.175 | 30 |  |
| Lipid Metabolism, Small Molecule Biochemistry, Vitamin and Mineral Metabolism | metabolism of sterol | 5.88E-08 | Decreased | -2.565 | 15 |  |
| Lipid Metabolism, Small Molecule Biochemistry, Vitamin and Mineral Metabolism | metabolism of cholesterol | 2.47E-07 | Decreased | -2.376 | 14 |  |
| Organismal Survival | organismal death | 7.08E-07 | Increased | 4.143 | 92 |  |
| Cell Cycle, Cellular Movement | cytokinesis of tumor cell lines | 3.36E-06 | Decreased | -2.357 | 10 |  |
| Cell Cycle, Reproductive System Development and Function | entry into interphase of oocytes | 3.90E-06 | Decreased | -2 | 4 |  |
| Cell Death and Survival | cell survival | 3.95E-06 | Decreased | -3.811 | 60 |  |
| Cell Death and Survival | cell viability | 3.51E-05 | Decreased | -3.074 | 54 |  |
| Lipid Metabolism, Small Molecule Biochemistry, Vitamin and Mineral Metabolism | steroid metabolism | 5.71E-05 | Decreased | -2.723 | 18 |  |
| Cellular Development, Cellular Growth and Proliferation | proliferation of hepatoma cell lines | 1.48E-04 | Decreased | -2.867 | 14 | |
| Cell Death and Survival | cell viability of tumor cell lines | 1.84E-04 | Decreased | -3.401 | 35 | |
| Cancer, Gastrointestinal Disease, Hepatic System Disease, Organismal Injury and Abnormalities | growth of liver tumor | 3.37E-04 | Decreased | -2.599 | 7 | |
| Cancer, Gastrointestinal Disease, Hepatic System Disease, Organismal Injury and Abnormalities | growth of hepatocellular carcinoma | 4.45E-04 | Decreased | -2.217 | 5 | |
| Cancer, Organismal Injury and Abnormalities | head and neck cancer | 1.09E-03 | Increased | 2.183 | 29 | |
| Cell Morphology, Cellular Function and Maintenance | repair of cells | 1.21E-03 | Decreased | -2.818 | 8 | |
| Cell Death and Survival | cell viability of myeloma cell lines | 1.50E-03 | Decreased | -2.828 | 8 | |
| Embryonic Development, Organismal Survival | death of embryo | 1.50E-03 | Increased | 3.293 | 11 | |
| Cellular Development, Cellular Growth and Proliferation | proliferation of carcinoma cell lines | 2.21E-03 | Decreased | -2.179 | 17 | |
| Infectious Disease | Viral Infection | 2.44E-03 | Decreased | -3.828 | 57 | |
| Cell Morphology, Cellular Function and Maintenance | repair of tumor cell lines | 2.48E-03 | Decreased | -2.449 | 6 | |
| Tissue Morphology | quantity of tumor cell lines | 2.49E-03 | Increased | 2.241 | 10 | |
| Cancer, Gastrointestinal Disease, Organismal Injury and Abnormalities | growth of digestive organ tumor | 3.02E-03 | Decreased | -2.773 | 8 | |
| Cell Cycle | aneuploidy | 4.08E-03 | Increased | 2.4 | 7 | |
| Infectious Disease, Organismal Injury and Abnormalities | infection of embryonic cell lines | 9.10E-03 | Decreased | -2.365 | 14 | |
| Infectious Disease | infection of epithelial cell lines | 9.10E-03 | Decreased | -2.365 | 14 | |
| Cancer, Organismal Injury and Abnormalities | growth of carcinoma | 9.52E-03 | Decreased | -2.938 | 9 | |
| Infectious Disease, Renal and Urological Disease | infection of kidney cell lines | 1.14E-02 | Decreased | -2.273 | 14 | |
| Cellular Development, Cellular Growth and Proliferation | proliferation of breast cancer cell lines | 1.32E-02 | Decreased | -2.89 | 18 | |
