# Supplementary material for: “Stockpile” of Slight Transcriptomic Changes Determines the Indirect Genotoxicity of Low-Dose BPA in Thyroid Cells
Source: PLoS One. 2016 Mar 16;11(3):e0151618. doi: 10.1371/journal.pone.0151618 (PMC4794173; doi:10.1371/journal.pone.0151618)
Supplement: S3 Table — IPA biofunctions with a significant activation state prediction are reported. IPA z-score predicts the effect of gene expression changes on significantly enriched biological functions. The activation state of a function is predicted increased for z-score ≥2 and decreased for z-score ≤-2. (DOCX) [file pone.0151618.s007.docx]

**S3 Table.** IPA biofunctions deregulated following 7-day BPA treatment in FRTL-5 cells

| Categories | Diseases or Functions Annotation | *p*-Value | Predicted Activation State | Activation *z*-score | Number of molecules |
| --- | --- | --- | --- | --- | --- |
| Infectious Disease | viral infection | 3.72E-05 | Decreased | -7.04 | 151 |
| Cellular Growth and Proliferation | proliferation of cells | 1.19E-04 | Decreased | -5.59 | 291 |
| Infectious Disease | infection of tumor cell lines | 1.25E-04 | Decreased | -7.01 | 59 |
| Gene Expression | transactivation | 2.79E-04 | Decreased | -2.42 | 64 |
| Gene Expression | transactivation of RNA | 2.79E-04 | Decreased | -2.24 | 61 |
| Infectious Disease | infection of cells | 3.13E-04 | Decreased | -7.74 | 81 |
| Infectious Disease | infection by HIV-1 | 6.32E-04 | Decreased | -7.40 | 64 |
| Infectious Disease | infection by RNA virus | 7.88E-04 | Decreased | -8.01 | 84 |
| Infectious Disease, Reproductive System Disease | infection of cervical cancer cell lines | 1.59E-03 | Decreased | -6.61 | 48 |
| Embryonic Development | size of embryo | 4.10E-03 | Decreased | -5.93 | 36 |
| Organismal Development | size of animal | 4.97E-03 | Decreased | -4.37 | 23 |
| Infectious Disease | infection by lentivirus | 5.92E-03 | Decreased | -7.27 | 67 |
| Cell Death and Survival | cell viability of cervical cancer cell lines | 5.92E-03 | Decreased | -4.20 | 25 |
| Infectious Disease | HIV infection | 8.93E-03 | Decreased | -7.40 | 66 |
| Post-Translational Modification | ubiquitination of protein | 1.24E-02 | Decreased | -2.27 | 27 |
| Cell Death and Survival | cell viability of tumor cell lines | 2.13E-02 | Decreased | -5.59 | 66 |
| Cell Death and Survival | cell death | 4.70E-03 | Increased | 3.14 | 259 |
| Cancer | carcinoma | 4.80E-02 | Increased | 2.36 | 431 |
| Organismal Survival | organismal death | 4.80E-02 | Increased | 12.28 | 178 |
|  |  |  |  |  |  |
