# Supplementary material for: “Stockpile” of Slight Transcriptomic Changes Determines the Indirect Genotoxicity of Low-Dose BPA in Thyroid Cells
Source: PLoS One. 2016 Mar 16;11(3):e0151618. doi: 10.1371/journal.pone.0151618 (PMC4794173; doi:10.1371/journal.pone.0151618)
Supplement: S4 Table — The 10 higher scored functional networks are listed, with the relative score and the number of molecules belonging to the network. (DOCX) [file pone.0151618.s008.docx]

**S4 Table.** Top 10 IPA associated functional networks modulated by 3-day BPA treatment in FRTL-5 cells

| Top Diseases and Functions | Score | Focus molecules |
| --- | --- | --- |
| DNA Replication, Recombination, and Repair, Cell Cycle, Cellular Movement | 52 | 30 |
| Cell Cycle, Cellular Assembly and Organization, DNA Replication, Recombination, and Repair | 45 | 27 |
| DNA Replication, Recombination, and Repair, Cell Cycle, Cellular Assembly and Organization | 45 | 27 |
| Cell Cycle, Cellular Assembly and Organization, DNA Replication, Recombination, and Repair | 45 | 27 |
| Infectious Disease, Dermatological Diseases and Conditions, Immunological Disease | 40 | 25 |
| Lipid Metabolism, Small Molecule Biochemistry, Vitamin and Mineral Metabolism | 38 | 24 |
| Endocrine System Development and Function, Lipid Metabolism, Small Molecule Biochemistry | 33 | 22 |
| Drug Metabolism, Glutathione Depletion In Liver, Cellular Assembly and Organization | 31 | 21 |
| Cancer, Hematological Disease, Immunological Disease | 31 | 21 |
| Metabolic Disease, Neurological Disease, Ophthalmic Disease | 25 | 18 |
