# Supplementary material for: “Stockpile” of Slight Transcriptomic Changes Determines the Indirect Genotoxicity of Low-Dose BPA in Thyroid Cells
Source: PLoS One. 2016 Mar 16;11(3):e0151618. doi: 10.1371/journal.pone.0151618 (PMC4794173; doi:10.1371/journal.pone.0151618)
Supplement: S5 Table — The 10 higher scored functional networks are listed, with the relative score and the number of molecules belonging to the network. (DOCX) [file pone.0151618.s009.docx]

**S5 Table.** Top 10 IPA associated functional networks modulated by 7-day BPA treatment in FRTL-5 cells

| Top Diseases and Functions | Score | Focus molecules |
| --- | --- | --- |
| Auditory Disease, Dermatological Diseases and Conditions, Developmental Disorder | 51 | 35 |
| DNA Replication, Recombination, and Repair, Developmental Disorder, Hereditary Disorder | 48 | 34 |
| Developmental Disorder, Hereditary Disorder, Inflammatory Disease | 43 | 32 |
| Carbohydrate Metabolism, Cell Morphology, Cellular Assembly and Organization | 43 | 32 |
| Carbohydrate Metabolism, Lipid Metabolism, Small Molecule Biochemistry | 38 | 30 |
| Amino Acid Metabolism, Small Molecule Biochemistry, Drug Metabolism | 38 | 30 |
| Cell Cycle, Protein Synthesis, Molecular Transport | 38 | 30 |
| Gene Expression, Protein Synthesis, Tissue Morphology | 36 | 29 |
| Gene Expression, Neurological Disease, Hereditary Disorder | 36 | 29 |
| Cell Cycle, DNA Replication, Recombination, and Repair, Infectious Disease | 34 | 28 |
