# Supplementary material for: “Stockpile” of Slight Transcriptomic Changes Determines the Indirect Genotoxicity of Low-Dose BPA in Thyroid Cells
Source: PLoS One. 2016 Mar 16;11(3):e0151618. doi: 10.1371/journal.pone.0151618 (PMC4794173; doi:10.1371/journal.pone.0151618)
Supplement: S6 Table — The union of genes enriched in the “DNA replication, recombination and repair” network at 3 and 7 days is reported with respective FCs and p-values. Significant FCs and p-values are in bold. (DOCX) [file pone.0151618.s010.docx]

**S6 Table.** Genes in the “DNA replication, recombination and repair” network predicted deregulated after 3- and 7-day exposure to BPA.

| **Gene name** | **Gene description** | **FC** | ***p*-value** | **FC** | ***p*-value** |
| --- | --- | --- | --- | --- | --- |
|  |  | **3 days** | | **7 days** | |
| *Abcc4* | ATP-binding cassette, sub-family C (CFTR/MRP), member 4 | **2.63** | **1.53E-03** | -1.75 | 1.54E-04 |
| *Adsl* | adenylosuccinate lyase | -1.35 | 5.97E-03 | **-2.36** | **3.26E-04** |
| *Ankfy1* | ankyrin repeat and FYVE domain containing 1 | -1.04 | 7.67E-03 | **-2.44** | **2.86E-03** |
| *Arpc3* | actin related protein 2/3 complex, subunit 3 | -1.43 | 8.57E-04 | **-2.05** | **3.96E-05** |
| *Btbd1* | BTB (POZ) domain containing 1 | -1.10 | 1.74E-03 | **-2.11** | **6.55E-04** |
| *Casp4* | caspase 4, apoptosis-related cysteine peptidase | 2.42 | 1.76E-03 | **-2.24** | **1.08E-04** |
| *Cops4* | COP9 constitutive photomorphogenic homolog subunit 4 | -1.50 | 1.23E-03 | **-2.13** | **1.15E-03** |
| *Cops6* | COP9 constitutive photomorphogenic homolog subunit 6 | -1.23 | 1.08E-02 | **-2.36** | **7.48E-03** |
| *Cops8* | COP9 constitutive photomorphogenic homolog subunit 8 | -1.30 | 9.67E-03 | **-2.39** | **1.61E-03** |
| *Creld2* | cysteine-rich with EGF-like domains 2 | **2.20** | **5.81E-05** | -1.54 | 7.73E-06 |
| *Cstb* | cystatin B (stefin B) | **-2.55** | **2.11E-03** | **-2.35** | **1.70E-03** |
| *Cul4a* | cullin 4A | -1.06 | 9.33E-03 | **-2.50** | **1.26E-04** |
| *Dcaf10* | DDB1 and CUL4 associated factor 10 | 1.08 | 1.08E-01 | **-2.02** | **5.52E-03** |
| *Dcaf4* | DDB1 and CUL4 associated factor 4 | -1.24 | 1.01E-01 | **-2.30** | **1.79E-03** |
| *Dctn5* | dynactin 5 | **-2.04** | **4.98E-04** | **-2.35** | **1.12E-03** |
| *Dctpp1* | dCTP pyrophosphatase 1 | **-2.16** | **4.08E-04** | -1.35 | 1.95E-04 |
| *Dda1* | DET1 and DDB1 associated 1 | **-1.64** | **9.95E-04** | **-2.57** | **6.49E-04** |
| *Ddb1* | damage-specific DNA binding protein 1 | -1.14 | 1.04E-01 | **-2.16** | **7.72E-03** |
| *Ect2* | epithelial cell transforming sequence 2 oncogene | **-4.68** | **6.44E-06** | -1.08 | 1.82E-05 |
| *Edaradd* | EDAR-associated death domain | -1.35 | 1.28E-02 | **-2.09** | **3.39E-03** |
| *Erap1* | endoplasmic reticulum aminopeptidase 1 | -1.27 | 1.22E-02 | **-2.09** | **2.21E-02** |
| *Fbxw4* | F-box and WD repeat domain containing 4 | 1.02 | 6.56E-01 | **-2.06** | **2.62E-02** |
| *Fem1b* | fem-1 homolog b | 1.51 | 3.54E-03 | **-2.04** | **3.38E-02** |
| *Herpud1* | homocysteine-inducible, endoplasmic reticulum stress-inducible, ubiquitin-like domain member 1 | 2.21 | 2.27E-05 | **-2.33** | **2.03E-06** |
| *Hivep2* | human immunodeficiency virus type I enhancer binding protein 2 | 1.12 | 1.15E-01 | **-2.10** | **3.25E-03** |
| *Hmgcs1* | 3-hydroxy-3-methylglutaryl-Coenzyme A synthase 1 (soluble) | **-4.92** | **7.43E-07** | -1.74 | 7.05E-07 |
| *Hsd17b10* | hydroxysteroid (17-beta) dehydrogenase 10 | **-2.03** | **8.10E-05** | -1.98 | 2.72E-04 |
| *Ibtk* | inhibitor of Bruton agammaglobulinemia tyrosine kinase | -1.06 | 8.79E-01 | **-2.15** | **8.02E-03** |
| *Idh1* | isocitrate dehydrogenase 1 (NADP+), soluble | **-2.86** | **2.94E-04** | -1.36 | 2.46E-04 |
| *Kdm2a* | lysine (K)-specific demethylase 2A | -1.08 | 5.83E-02 | **-2.00** | **6.47E-03** |
| *Kif23* | kinesin family member 23 | **-2.94** | **7.69E-07** | 1.09 | 7.05E-07 |
| *Klhl22* | kelch-like 22 | 1.01 | 7.08E-02 | **-2.28** | **9.69E-03** |
| *Lta4h* | leukotriene A4 hydrolase | -1.55 | 1.86E-04 | **-2.42** | **5.95E-04** |
| *Mdh1* | malate dehydrogenase 1, NAD (soluble) | -1.88 | 7.13E-04 | **-2.33** | **7.37E-04** |
| *Myo1e* | myosin IE | 1.12 | 2.61E-01 | **-2.07** | **1.95E-03** |
| *Nans* | N-acetylneuraminic acid synthase | **-2.11** | **6.11E-03** | **-3.16** | **2.45E-03** |
| *Pak1ip1* | PAK1 interacting protein 1 | **-2.23** | **1.37E-06** | **-2.01** | **5.69E-06** |
| *Plk1* | polo-like kinase 1 | **-5.95** | **2.11E-06** | -1.12 | 6.56E-06 |
| *Plk4* | polo-like kinase 4 | **-2.34** | **5.37E-05** | -1.17 | 4.08E-05 |
| *Prc1* | protein regulator of cytokinesis 1 | **-3.84** | **1.67E-06** | -1.68 | 2.81E-06 |
| *Psma6* | proteasome (prosome, macropain) subunit, alpha type 6 | **-2.59** | **1.29E-04** | **-2.12** | **1.09E-04** |
| *Psmb3* | proteasome (prosome, macropain) subunit, beta type 3 | **-2.16** | **7.30E-05** | -1.60 | 1.02E-04 |
| *RGD1310444* | LOC363015 | **-2.52** | **6.83E-04** | **-2.95** | **5.63E-03** |
| *Racgap1* | Rac GTPase-activating protein 1 | **-3.27** | **1.34E-06** | -1.20 | 1.97E-06 |
| *Sc4mol* | sterol-C4-methyl oxidase-like | **-3.44** | **7.87E-05** | -1.20 | 5.73E-05 |
| *Scly* | selenocysteine lyase | -1.12 | 9.01E-01 | **-2.34** | **7.25E-03** |
| *Slc3a2* | solute carrier family 3 (activators of dibasic and neutral amino acid transport), member 2 | **2.32** | **8.36E-05** | -1.26 | 5.30E-05 |
| *Slc5a5* | solute carrier family 5 (sodium iodide symporter), member 5 | **2.17** | **2.88E-02** | -1.47 | 1.39E-01 |
| *Slc6a9* | solute carrier family 6 (neurotransmitter transporter, glycine), member 9 | **3.74** | **7.99E-06** | -1.14 | 3.91E-06 |
| *Slc7a11* | solute carrier family 7 (cationic amino acid transporter, y+ system), member 11 | **10.22** | **7.43E-07** | 1.18 | 7.05E-07 |
| *Smc2* | structural maintenance of chromosomes 2 | **-3.17** | **4.04E-04** | 1.07 | 1.65E-04 |
| *Smc4* | structural maintenance of chromosomes 4 | **-2.57** | **4.38E-05** | -1.14 | 1.33E-04 |
| *Taok2* | TAO kinase 2 | 1.08 | 7.78E-02 | **-2.29** | **3.65E-04** |
| *Topbp1* | topoisomerase (DNA) II binding protein 1 | **-2.22** | **3.04E-06** | -1.47 | 1.64E-05 |
| *Trpc4ap* | transient receptor potential cation channel, subfamily C, member 4 associated protein | -1.42 | 1.54E-02 | **-2.64** | **2.67E-04** |
| *Uhmk1* | U2AF homology motif (UHM) kinase 1 | 1.01 | 6.70E-02 | **2.16** | **2.87E-02** |
| *Vprbp* | Vpr (HIV-1) binding protein | -1.07 | 7.74E-03 | **-2.19** | **1.32E-03** |
| *Wdtc1* | WD and tetratricopeptide repeats 1 | 1.39 | 4.53E-02 | **-2.01** | **5.75E-03** |
| *Zmym4* | zinc finger, MYM-type 4 | 1.03 | 4.59E-01 | **-2.06** | **2.77E-02** |
| *Zyg11b* | zyg-11 homolog B | -1.08 | 6.91E-02 | **-2.15** | **3.06E-03** |
