# Supplementary material for: “Stockpile” of Slight Transcriptomic Changes Determines the Indirect Genotoxicity of Low-Dose BPA in Thyroid Cells
Source: PLoS One. 2016 Mar 16;11(3):e0151618. doi: 10.1371/journal.pone.0151618 (PMC4794173; doi:10.1371/journal.pone.0151618)
Supplement: S7 Table — The union of genes enriched in the “checkpoint control” category at 3 and 7 days are reported with respective FCs and p-values. Significant FCs and p-values are in bold. (DOCX) [file pone.0151618.s011.docx]

**S7 Table.** Genes in the “checkpoint control” category predicted deregulated after 3- and 7-day exposure to BPA.

| **Gene name** | **Gene description** | **FC** | ***p*-value** | **FC** | ***p*-value** |
| --- | --- | --- | --- | --- | --- |
|  |  | **3 days** | | **7 days** | |
| *Bid* | BH3 interacting domain death agonist | -1.37 | 3.03E-04 | **-2.43** | **3.54E-05** |
| *Bub1* | budding uninhibited by benzimidazoles 1 homolog | **-4.54** | **5.06E-06** | -1.02 | 1.35E-05 |
| *Bub1b* | budding uninhibited by benzimidazoles 1 homolog, beta | **-2.21** | **1.30E-05** | -1.10 | 6.51E-06 |
| *Ccnb1* | cyclin B1 | **-5.78** | **1.75E-07** | -1.46 | 5.79E-07 |
| *Ccnb2* | cyclin B2 | **-4.31** | **1.17E-06** | -1.07 | 5.04E-06 |
| *Ccne2* | cyclin E2 | **-3.61** | **4.39E-06** | -1.31 | 6.63E-06 |
| *Ccng2* | cyclin G2 | -1.01 | 2.46E-02 | **-2.10** | **2.14E-04** |
| *Cdc20* | cell division cycle 20 homolog | **-5.46** | **1.07E-05** | -1.46 | 1.22E-05 |
| *Chfr* | checkpoint with forkhead and ring finger domains | -1.20 | 5.87E-05 | **-2.36** | **7.27E-06** |
| *Cks1b* | CDC28 protein kinase regulatory subunit 1B | **-3.68** | **4.50E-04** | **-2.04** | **6.21E-04** |
| *Csnk1g1* | casein kinase 1, gamma 1 | 1.12 | 7.17E-02 | **-2.20** | **2.32E-03** |
| *Cul4a* | cullin 4A | -1.06 | 9.33E-03 | **-2.50** | **1.26E-04** |
| *Fancd2* | Fanconi anemia, complementation group D2 | **-2.39** | **1.17E-06** | -1.50 | 1.96E-06 |
| *Gtf2h5* | general transcription factor IIH, polypeptide 5 | -1.43 | 1.53E-04 | **-2.06** | **5.41E-05** |
| *Hmgn1* | high-mobility group nucleosome binding domain 1 | -1.08 | 1.28E-02 | **2.02** | **5.40E-04** |
| *Mad2l1* | MAD2 mitotic arrest deficient-like 1 | **-3.11** | **1.05E-05** | -1.01 | 7.27E-06 |
| *Mcm7* | minichromosome maintenance complex component 7 | **-3.71** | **8.09E-06** | -1.54 | 1.94E-05 |
| *Mdm2* | MDM2 proto-oncogene, E3 ubiquitin protein ligase | 1.15 | 7.66E-02 | **-2.13** | **2.62E-04** |
| *Ndc80* | NDC80 homolog, kinetochore complex component | **-2.17** | **2.29E-05** | 1.02 | 1.06E-05 |
| *Obfc2a* | oligonucleotide/oligosaccharide-binding fold containing 2A | -1.40 | 8.96E-04 | **-2.01** | **1.09E-04** |
| *Plk1* | polo-like kinase 1 | **-5.95** | **2.11E-06** | -1.12 | 6.56E-06 |
| *Pttg1* | pituitary tumor-transforming 1 | **-4.58** | **1.60E-06** | 1.02 | 1.51E-06 |
| *RGD1562582* | similar to KIAA0406-like protein | 1.16 | 1.57E-02 | **-2.09** | **2.81E-03** |
| *Rad18* | RAD18 homolog | **-2.05** | **2.29E-06** | -1.56 | 4.03E-06 |
| *Trrap* | transformation/transcription domain-associated protein | -1.03 | 8.32E-03 | **-2.09** | **7.62E-04** |
| *Uimc1* | ubiquitin interaction motif containing 1 | -1.25 | 5.97E-03 | **-2.06** | **6.73E-04** |
| *Zwilch* | zwilch kinetochore protein | **-2.39** | **4.31E-06** | **-2.02** | **1.17E-05** |
