# Supplementary material for: “Stockpile” of Slight Transcriptomic Changes Determines the Indirect Genotoxicity of Low-Dose BPA in Thyroid Cells
Source: PLoS One. 2016 Mar 16;11(3):e0151618. doi: 10.1371/journal.pone.0151618 (PMC4794173; doi:10.1371/journal.pone.0151618)
Supplement: S8 Table — IPA overlap p-values (Fisher's exact test) are used to assess whether there is a statistically significant overlap between genes in the data set and genes regulated by a specific transcriptional regulator. The IPA z-score predicts the effect of gene expression changes on significantly predicted transcriptional regulators. The activation state of a transcriptional regulator is predicted activated for z-score ≥2 and inhibited for z-score ≤-2. (DOCX) [file pone.0151618.s012.docx]

**S8 Table.** Top IPA predicted transcription regulators after 7-day treatment with 10^-9^ M BPA in FRTL-5 cells

| Transcription Regulator | Predicted Activation State | Regulation *z*-score | *p*-value of overlap |
| --- | --- | --- | --- |
| MYC | Inhibited | -3.426 | 5.36E-10 |
| TP53 | Inhibited | -4.281 | 1.36E-08 |
| ATF4 | Inhibited | -3.24 | 2.35E-05 |
| XBP1 | Inhibited | -4.146 | 5.10E-05 |
| FOXO3 | Inhibited | -2.16 | 2.55E-04 |
| NFE2L2 | Inhibited | -4.731 | 5.56E-04 |
| PDX1 | Activated | 2.673 | 5.90E-04 |
| NRF1 | Inhibited | -2.449 | 2.80E-03 |
| STAT4 | Inhibited | -4.08 | 6.21E-03 |
| SMARCA4 | Inhibited | -2.954 | 6.45E-03 |
| FOXO1 | Inhibited | -2.505 | 8.30E-03 |
| TP73 | Inhibited | -3.011 | 1.57E-02 |
