# Supplementary material for: “Stockpile” of Slight Transcriptomic Changes Determines the Indirect Genotoxicity of Low-Dose BPA in Thyroid Cells
Source: PLoS One. 2016 Mar 16;11(3):e0151618. doi: 10.1371/journal.pone.0151618 (PMC4794173; doi:10.1371/journal.pone.0151618)
Supplement: S9 Table — Genes whose deregulation has been verified by qRT-PCR are listed. For each of them, the gene description, the fold change and relative p-value of both qRT-PCR and microarray experiments are reported. (DOCX) [file pone.0151618.s013.docx]

**S9 Table.** Microarray validation of genes deregulated in the 7-day data set by qRT-PCR

|  |  | qRT-PCR | | microarray | |
| --- | --- | --- | --- | --- | --- |
| Gene name | Gene description | FC | *p-*value | FC | *p-*value |
| *Atf4* | activating transcription factor 4 | -2.25 | 9.68E-03 | -2.02 | 1.23E-05 |
| *Bid* | BH3 interacting domain death agonist | -2.32 | 9.88E-03 | -2.43 | 1.13E-05 |
| *Ddit3* | DNA-damage inducible transcript 3 | -2.17 | 5.12E-03 | -2.49 | 3.00E-05 |
| *E2f5* | E2F transcription factor 5 | -1.68 | 1.12E-02 | -2.77 | 1.07E-04 |
| *Ddb1* | damage-specific DNA binding protein 1 | -1.54 | 1.39E-05 | -2.16 | 2.47E-03 |
| *Cops5* | COP9 constitutive photomorphogenic homolog subunit 5 | -1.68 | 2.5E-04 | -2.23 | 1.34E-05 |
| *Cops4* | COP9 constitutive photomorphogenic homolog subunit 4 | -1.52 | 5.32E-04 | -2.13 | 3.10E-04 |
| *Cops6* | COP9 constitutive photomorphogenic homolog subunit 6 | -1.81 | 1.07E-03 | -2.36 | 2.38E-03 |
| *Cops8* | COP9 constitutive photomorphogenic homolog subunit 8 | -1.63 | 6.06E-04 | -2.39 | 4.46E-04 |
| *SerpinB9* | serine (or cysteine) peptidase inhibitor, clade B, member 9 | -1.5 | 1.98E-03 | -2.6 | 7.04E-05 |
| *Mdm4* | MDM4, p53 regulator | -1.6 | 1.60E-03 | -2.33 | 1.79E-02 |
| *Gclm* | glutamate-cysteine ligase, modifier subunit | -1.5 | 9.56E-03 | -2.33 | 1.79E-02 |
| *Id3* | inhibitor of DNA binding 3 | -1.4 | 1.13E-02 | -2.44 | 2.70E-04 |
| *Cat* | catalase | -1.6 | 8.82E-03 | -2.06 | 4.26E-03 |
| *Vprbp* | Vpr (HIV-1) binding protein | -1.64 | 9.16E-03 | -2.19 | 3.57E-04 |
| *Irf3* | interferon regulatory factor 3 | -1.82 | 1.03E-02 | -2.59 | 1.42E-04 |
| *Fem1b* | fem-1 homolog b | -1.56 | 1.52E-02 | -2.03 | 1.43E-02 |
| *Wdtc1* | WD and tetratricopeptide repeats 1 | -1.57 | 2.23E-03 | -2.01 | 1.78E-03 |
| *Smad6* | SMAD family member 6 | -1.74 | 1.45E-02 | -2.20 | 2.88E-04 |
